# Common Photoproperties of Eumelanin and Natural Organic Matter Emerge from Ensembles of Few-Layered Nanostructures

**DOI:** 10.1021/acscentsci.5c02304

**Published:** 2026-04-16

**Authors:** Meera Madhu, Aleksandra Ilina, Hang Li, Garrett McKay, Bern Kohler

**Affiliations:** † Department of Chemistry and Biochemistry, 2647 The Ohio State University, Columbus, Ohio 43210, United States; ‡ Zachry Department of Civil and Environmental Engineering, Texas A&M University, College Station, Texas 77843, United States

## Abstract

Carbon-based nanomaterials play central roles in both natural systems and emerging sustainable technologies, serving diverse functions from photoprotection in living organisms to energy harvesting and storage. Eumelanin, the brown-black melanin pigment, and natural organic matter (NOM), a similarly colored substance formed from the decomposition of biological material, represent two of the most widespread yet structurally elusive carbon-based materials in nature. Here, we present a detailed steady-state and time-resolved spectroscopic study, which reveals deep similarities in their photophysical properties as a consequence of common structural motifs at the nanoscale. While eumelanin and NOM have been traditionally studied independently and interpreted using molecular models, we introduce a framework centered on interchromophoric couplings to explain how common photoproperties emerge despite differences in their atomistic structures. By disassembling hierarchically structured eumelanin nanoparticles, we identify the fundamental spectroscopic units to be *π*-stacked aggregates consisting of only a few layers. Our model explains how common photoproperties including transient spectral hole burning and excitation-wavelength-dependent emission arise from the ensemble behavior of these units. Our findings underscore the importance of focusing on how properties evolve through different levels of hierarchical assembly and provide a foundation for a unified photophysical framework that spans natural and lab-made carbon-based nanomaterials.

## Graphical abstract

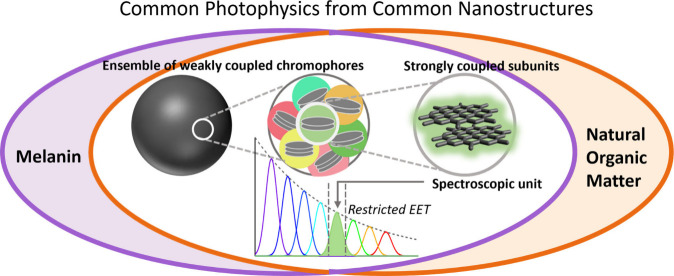

## 1 Introduction

Eumelanin and natural organic matter (NOM) appear at first glance to have little in common beyond their brown colors. The black-brown eumelanin, which is one of five classes of natural melanin pigments, provides pigmentation in countless organisms including humans,^1,2^ while NOM is responsible for the yellow and brown hues seen in terrestrial waters and soils.^3–5^ Both NOM and melanin are ubiquitous in nature. Most of the reduced carbon in the biosphere is present as NOM,^6^ and the occurrence of melanin in nearly all organisms is a sign of the evolutionary significance of the pigment, which provides diverse biological functions that include camouflage, sunscreening, metal ion chelation, and radical scavenging.^7^

The starting materials and chemical pathways that lead to melanin and NOM are remarkably different. Melanin pigments are synthesized by living cells, while organic substances in NOM are derived from dead ones (Figure 1). In the case of eumelanin, a single chemical precursor, tyrosine, is transformed by bottom-up reactions with minimal involvement of enzymes into nanoscale granules that are brown-black in color.^8^ NOM, on the other hand, is similarly colored but is produced by top-down biotic and abiotic transformations of decaying plant and animal cells and has been shown to contain thousands of distinct chemical substances.^9,10^

**Figure 1:**
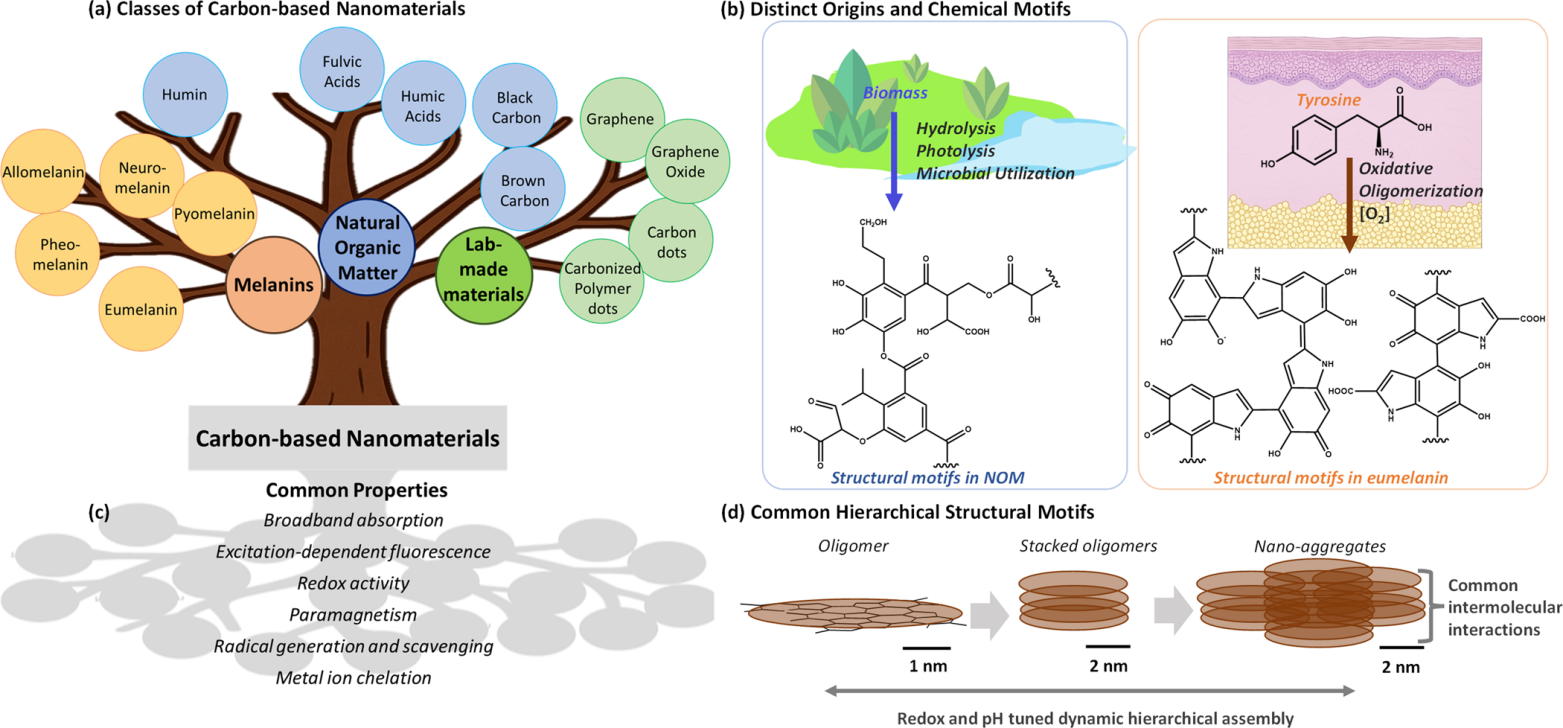
(a) Tree diagram showing relationships among natural and synthetic carbon-based materials. (b) Distinct origins and putative chemical motifs in natural organic matter (NOM) and eumelanin. (c) List of common properties of NOM and eumelanin. (d) Proposed common hierarchical structure, from oligomers to nanoaggregates.

Despite their different origins, eumelanin and NOM exhibit strikingly similar optical and other physicochemical properties (Figure 1c). These properties include structureless broadband absorption from UV to NIR wavelengths, low and monotonically decaying fluorescence quantum yields with increasing wavelength, enveloping fluorescence emission spectra,^5,7,11,12^ paramagnetism (stable radical populations),^13,14^ radical sourcing and scavenging capacity,^2,4^ and excellent metal ion chelation.^15,16^ Their similar properties allow melanin pigments and NOM to act analogously in their respective environments. For example, eumelanin pigments in mammalian skin inhibit DNA photodamage by limiting the penetration of solar UV radiation,^17,18^ while NOM in natural waters controls the penetration depth of light in the water column, screening organisms from photodamage.^19^

The common properties shared by eumelanin and NOM have been noted sporadically for decades.^11,20–26^ In the 1980s, Sarna, a melanin scientist, referred to humic acids as “melanin-like polymers”,^22^ while Senesi, a soil scientist, wrote of melanins as “humic acid-type polymers.”^23^ Despite intermittent statements in the literature about the relatedness of NOM and melanin, research has proceeded independently within the melanin and NOM communities. A literature review nevertheless reveals a history of parallel concepts and often nearly contemporaneous discoveries (Table 1), providing evidence of deep but underappreciated connections.

**Table 1:** Parallel Concepts in the Melanin and NOM Fields over the Years

	Melanin	NOM
Persistent free radicals	Commoner et al., 1954 ^27^	Rex, 1960 ^28^
Photogenerated radicals	Sever et al., 1962 ^29^	Senesi and Schnitzer, 1977 ^30^
Spin density increases with increasing pH	Sarna et al., 1980 ^22^	Wilson and Weber, 1977 ^31^
Excitation wavelength-dependent emission	Nighswander-Rempel et al., 2005 ^32^	Del Vecchio and Blough, 2004 ^11^
Quinhydrone-type CT complexes	Felix et al., 1978 ^33^	Steelink and Tollin, 1962 ^34^
Supramolecular structures	Cheng et al., 1994 ^35^	Piccolo, 2001 ^36^
Interacting chromophore models	Chen et al., 2014 ^37^	Del Vecchio and Blough, 2004 ^11^

Motivated by the desire to understand the common optical properties of eumelanin and NOM, this report has two aims. First, we draw attention to the remarkably similar optical properties of synthetic eumelanin and NOM through side-by-side measurements. Second, we advance the hypothesis that morphology and hierarchical structure (Figure 1d) are paramount for photoproperty emergence in melanin and NOM, pointing out the opportunity for a nanoscience perspective to advance understanding. The presence of common nanoscale interactions that support charge transfer (CT), excitonic coupling, and stable radical centers explain how common properties emerge in melanin and NOM despite differences in their molecular-level structures (Figure 1b).

The foundations for the unifying view that we promote here were laid in an elegant but obscure 1978 paper by Sławinski et al.,^21^ which illustrated melanins and humic and fulvic acids as branches in a tree of “carbonaceous polymers.” The authors included a third branch for oxygenated polycyclic aromatic hydrocarbons (PAHs) produced by pyrolysis. The latter category recognizes that melanin and NOM, which are synthesized at ambient temperatures, are similar to materials formed by incomplete combustion in air such as chars, coals, asphaltenes, and brown and black atmospheric carbon aerosols. We expand the tree of Sławinski et al., which was limited to materials found in the biosphere, to include a branch representing lab-made carbon-based nanomaterials like graphene oxide and carbon dots,^21^ which have similar properties (Figure 1a). Assembling data from NOM and eumelanin that can be used to develop models that explain optical and other properties across a broad family of related carbon-based nanomaterials is a further motivation of this study.

While researchers in each field have emphasized the importance of supramolecular interactions, there has been a strong tendency to view melanin and NOM through a “molecular lens,” regarding them as heterogeneous mixtures of weakly interacting molecules. For example, efforts to understand nonradiative decay in eumelanin have focused on excited-state proton transfer pathways observed in its putative monomer building blocks, 5,6-dihydroxyindole and 5,6-dihydroxyindole-2-carboxylic acid,^38–40^ assuming that deactivation pathways in these small molecules also operate in the nanomaterial. NOM, meanwhile, has frequently been conceptualized as heterogeneous mixtures of discrete molecular units.^41,42^ However, the particulate nature of melanin and NOM suggests that their molecular-scale substructures interact strongly with one another. Interactions that emerge at supramolecular length scales have received less attention in both fields. Augmenting a molecular-focused picture with one that emphasizes nanoscale interactions is required to understand the time-resolved and steady-state photoproperties of these and other carbon-based nanomaterials.

Here, we test the hypothesis that common supramolecular structures give rise to the common photoproperties of melanin and NOM by comparing their photophysics and characterizing the structures present in each using atomic force microscopy (AFM). Several NOM samples obtained from natural sources and synthetic eumelanin nanoparticles derived from the oxidative polymerization of L-DOPA (DOPAm) were investigated. Synthetic melanins offer greater reproducibility and control over morphology compared to natural melanin, which can vary significantly depending on the source, method of isolation, and the presence of residual proteins and bound metal ions.^1^ DOPAm is widely used as a model for natural eumelanin and is thought to capture its key chemical motifs and hierarchical structures.^43^ Similarities and differences between DOPAm and NOM samples are evaluated with time-resolved and steady-state methods, alkaline disassembly, and reduction with sodium borohydride.

Our study shows that similar steady-state photoproperties (i.e., absorption and emission) noted in two recent studies^25,26^ extend to time-domain observables: Transient spectral holes are observed when DOPAm or NOM samples are excited by femtosecond excitation pulses with visible wavelengths. We show that the photophysics of these materials are driven by their nanostructure by disassembling the synthetic eumelanin nanoparticles into ultrasmall (<5 nm) few-layered structures that closely mimic the optical properties of NOM samples obtained from aquatic and soil environments. Using AFM imaging, we show that the DOPAm and NOM samples contain abundant few-layer stacks of planar molecules, and we propose that these ultrasmall assemblies define the elementary chromophores whose ensemble-level behavior gives rise to their common photophysical properties. Taken together, this work advances understanding of how the optical properties of eumelanin and NOM are tuned by particle morphology and identifies length scales at which key optical properties emerge.

## 2 Results and Discussion

### 2.1 Steady-State and Transient Photoproperties of DOPAm and NOM

Steady-state and time-resolved spectroscopic measurements were performed on DOPAm (a synthetic eumelanin) and three NOM isolates: Elliott Soil Humic Acid (ESHA), Pahokee Peat Fulvic Acid (PPFA) and Suwannee River NOM (SRNOM). Full details about samples and sample preparation are in SI section S1. Hereafter, we will use the term native DOPAm to refer to the as-synthesized material to distinguish it from material that was modified by alkaline disassembly (section 2.3).

NOM and eumelanin differ in their atomistic structures as seen from their hypothesized chemical structures (Figure 1b), elemental composition (see Table S1 and SI Section S2), and infrared spectra (Figure 2c). Despite these compositional differences, NOM and eumelanin exhibit qualitatively similar steady-state and transient photoproperties as presented in this section. Nonetheless, quantitative differences in properties such as fluorescence quantum yields and excited-state lifetimes indicate that their common properties occur on a continuum. In later sections, we explore the hypothesis that these differences are tuned by nanoscale interactions among chromophores.

**Figure 2:**
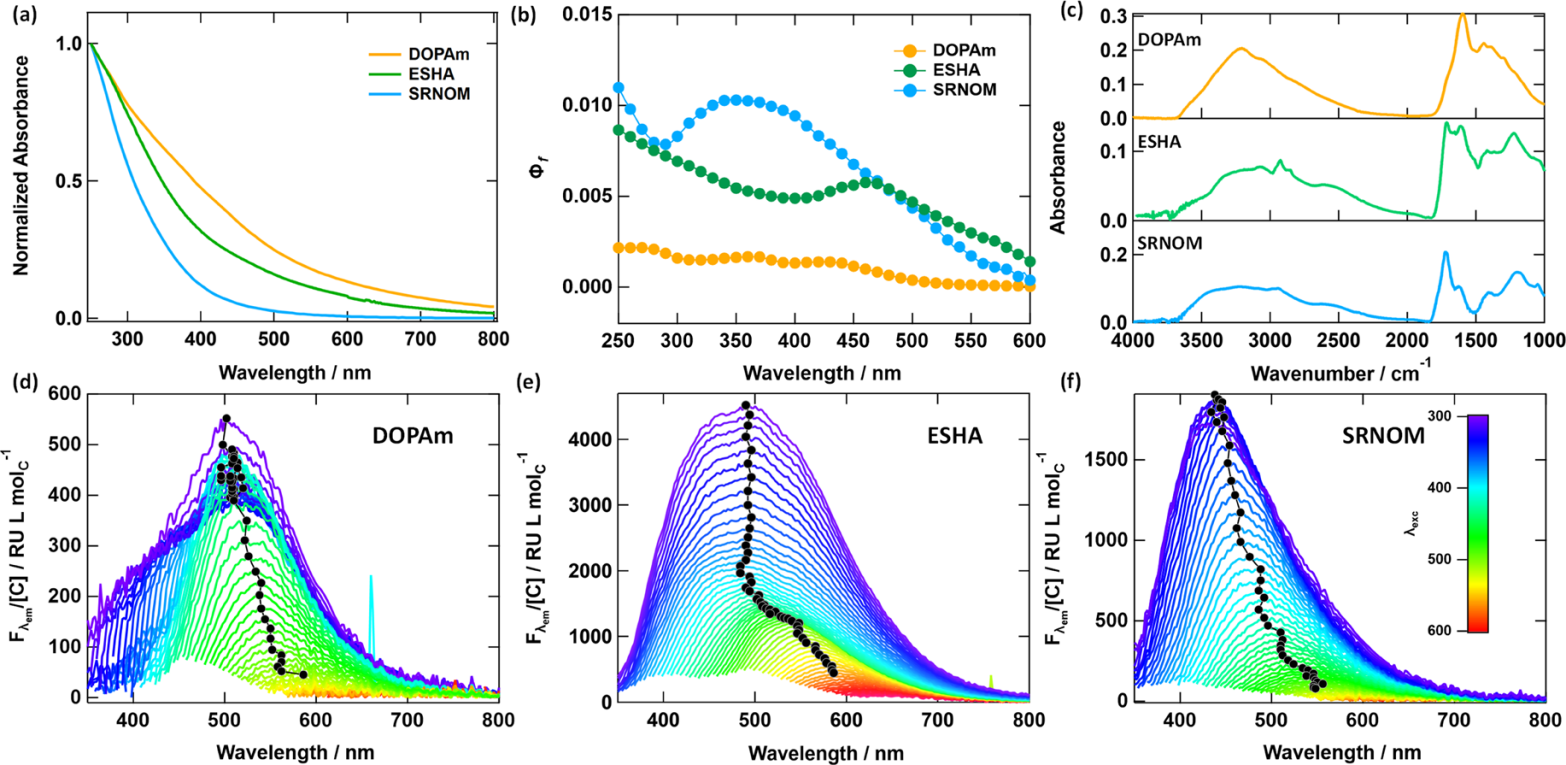
(a) UV–vis absorption spectra of DOPAm, ESHA, and SRNOM, normalized to have the same absorbance at 250 nm. (b) Fluorescence quantum yields for NOM and DOPAm as a function of excitation wavelength. (c) FTIR absorption spectra of NOM and DOPAm. Emission spectra of (d) DOPAm, (e) ESHA, and (f) SRNOM at different excitation wavelengths. The color map in panel f applies to all emission spectra. Black circles show the intensity-weighted emission maximum wavelength for each emission spectrum.

The electronic absorption spectra of both DOPAm and the NOM samples are broad, featureless, and decrease monotonically from UV to NIR wavelengths (Figure 2a). The spectra decay exponentially with wavelength such that a graph of log absorbance vs wavelength yields an approximately straight line. The slope of this line is nearly the same for PPFA and SRNOM but is smaller for ESHA and DOPAm (Figure S1, Table S2).

All DOPAm and NOM samples show weak fluorescence, but the fluorescence quantum yields (*Φ*_f_) of NOM are 5- to 10-fold higher than for DOPAm (Figures 2b, S2a). The *Φ*_f_ values increase in the order DOPAm < ESHA < SRNOM < PPFA. For both NOM and DOPAm, *Φ*_f_ decreases with increasing excitation wavelength. Likewise, the emission maximum wavelength (*λ*_em_^avg^, calculated as the intensity-weighted emission maximum, see SI section S5) (black circles in Figures 2d–f, S2b) remains constant at UV excitation wavelengths but increases with increasing excitation wavelength above a threshold wavelength that differs for each sample. The broadband, monotonically decaying absorption spectra and excitation wavelength-dependent emission (EWDE) have never been reproduced by a solution of a single molecular species but are signature photoproperties of synthetic eumelanin,^32^ NOM,^11^ and other carbon-based nanomaterials.^44,45^

Femtosecond transient absorption (TA) spectra recorded with excitation at 400 and 500 nm are shown for the native DOPAm and NOM samples in Figures 3a ***–*** f and S3, with data for 265 nm excitation in Figure S4. With 265 nm excitation, all samples exhibit broad photoinduced absorption (PIA) bands within the 350 ***–*** 620 nm probe window. The PIA bands for DOPAm and ESHA increase in amplitude as the probe wavelength increases, while the PIA from SRNOM and PPFA remains relatively flat through the probe window. For all samples, the kinetic traces decay in a biphasic fashion with a fast decay in the first few ps and a power-law decay evident ∼1 ns after excitation (Figure S5). Fitting parameters for the fits in Figure S5 are listed in Table S3 and fit residuals are shown in Figure S6. The fitting procedure is described in SI Section S6­(i).

**Figure 3:**
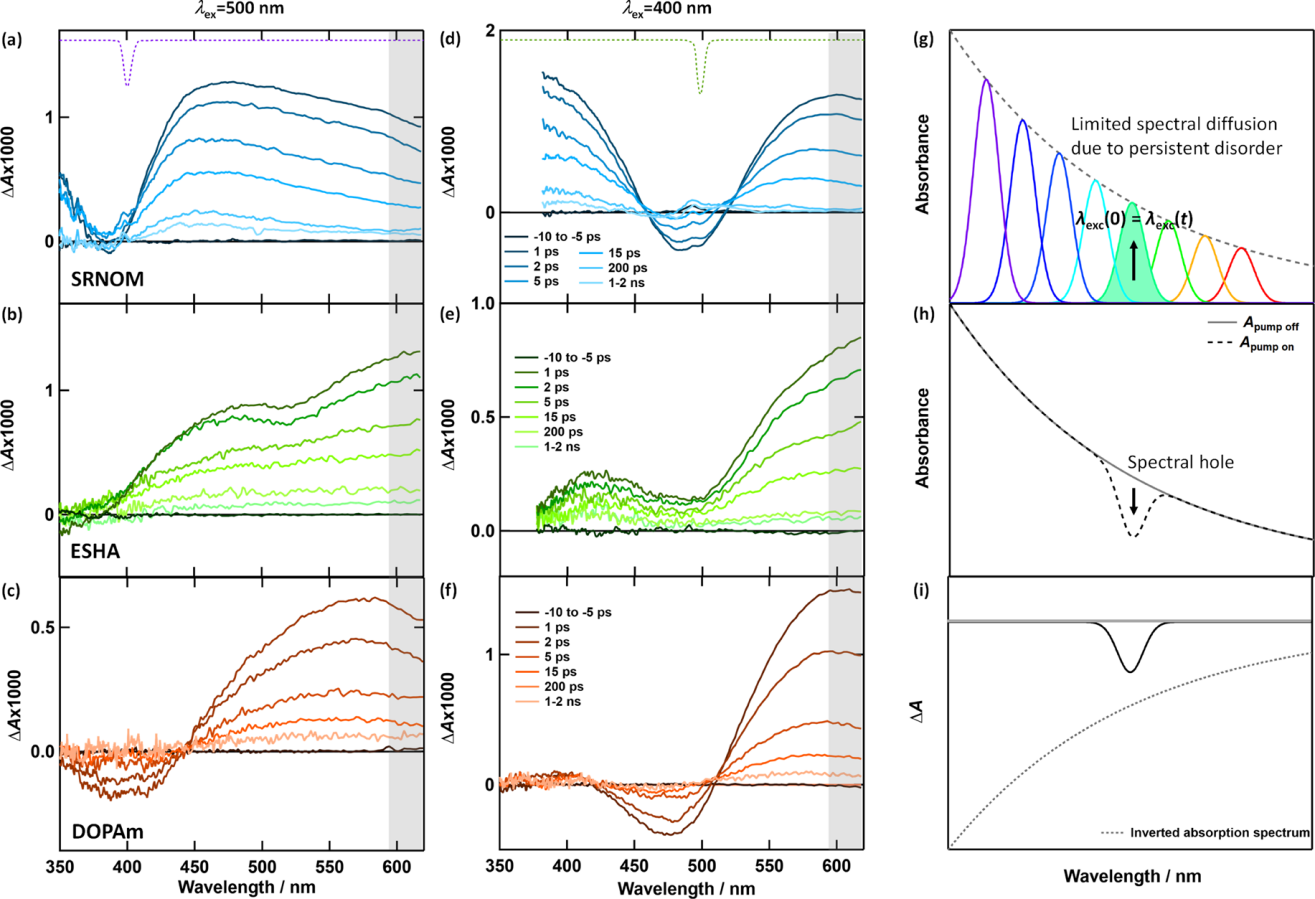
TA spectra of SRNOM, ESHA, and DOPAm recorded with excitation wavelengths of (a)–(c) 400 nm and (d)–(f) 500 nm. The inverted spectrum of the excitation pulse is shown at the top of panels a and d. The gray bar on the right edge of each graph shows the wavelength range used to obtain the average kinetic traces in Figure S5. (g) Schematic representation of subensembles of chromophores that absorb at different wavelengths due to chemical disorder (heterogeneity). (h) Absorption spectra measured with the pump on and off in transient spectral hole burning (TSHB) experiments. (i) Corresponding TA spectrum highlighting the appearance of the spectral hole as a negative absorbance change. In contrast to a single molecular substance, which bleaches uniformly over the full absorption spectrum, the signal here reflects selective bleaching of a subensemble of chromophores.

A broad dip or spectral hole located close to the center wavelength of the pump pulse is observed when the eumelanin and NOM samples are excited at visible wavelengths (Figures 3a–f, S3). Transient spectral hole burning (TSHB) has been observed previously in DOPAm^40,46,47^ but is reported here for the first time in NOM. The spectral holes are transient and decay to zero along with the PIA signals. Spectral hole profiles isolated following the procedure described in SI Section S6­(ii) are shown in Figure S7. The isolated spectral holes in the DOPAm and NOM samples are ∼0.5 eV wide (full width at half-maximum, fwhm, see Table S4), which is much greater than the bandwidth of the pump pulse, indicating that the dip in the spectrum is not an artifact due to scattering of the pump pulse. Furthermore, the hole widths are similar to features reported in steady-state photobleaching experiments on NOM by Del Vecchio and Blough,^11^ supporting the conclusion that the spectral holes arise from bleaching of chromophores.

### 2.2 TSHB and EWDE Arise from Restricted Energy Transfer

The observation of TSHB and EWDE provides insight into how the chromophores in NOM and eumelanin interact with each other and the surrounding environment. Here, we use “chromophore” to denote the portion of a system that is responsible for absorbing light of a given frequency. There is ambiguity in this concept, particularly for a supramolecular system or disordered solid in which couplings between molecular absorbers lead to delocalized excited states or excitons. Nonetheless, chromophore indicates approximately ‘how large’ an excited state is (i.e., it describes the region of a system that experiences a change in electron density as a result of excitation). Because excited states can change dramatically in size over their lifetime, we define a chromophore by the vertical or Franck–Condon excited state prior to relaxation. This agrees with usage in the conjugated polymer field where chromophores are the “primary absorbing units.”^48^

In TSHB, a pump laser with a bandwidth that is narrow compared to the sample’s absorption band (Figure 3a,d) excites a subensemble of chromophores that have transition frequencies in resonance with the pump laser. Due to photoselection, unexcited chromophores are depleted at the frequency of the pump laser (Figure 3h), allowing the spectral hole to be detected through diminished absorption (bleaching) (Figure 3i). Spectral diffusion (SD), arising from changes in the local environment of a chromophore, randomizes the transition energies of the unexcited chromophores, spreading them throughout the inhomogeneously broadened absorption band and filling in the spectral hole. Excitation energy transfer (EET) is another phenomenon that causes a similar randomization of transition energies. TSHB will only be observed if hole filling due to SD and EET occurs at significantly slower rates than excited state decay. For a system containing a single kind of chromophore, TSHB is typically only observed at the cryogenic temperatures needed to suppress environmental fluctuations.

TSHB occurs at ambient temperature in eumelanin and NOM because each contains chromophores that cannot undergo SD over the entire absorption spectrum. These chromophores are proposed to have different chemical structures, causing their transition energies to spread across the UV–vis–NIR, yielding the absorption spectrum of the full ensemble (Figure 3g). Like the unchanging local environments found at cryogenic temperatures, inhomogeneity in a chemically disordered sample is ‘frozen in’ as distinct chemical structures will always differ in their transition energies regardless of environmental changes. This restricts SD of individual chromophores to spectral ranges that are narrow compared to the broadband absorption profile.

The limit of fully uncoupled chromophores that absorb and emit at different wavelengths has been considered in both the NOM and melanin communities, where it is referred to as the superposition model^49–51^ or the chemical disorder model,^52,53^ respectively. However, these models cannot explain the excitation wavelength-dependent trends in excited state lifetimes, fluorescence quantum yields, or the constant width of spectral holes. For example, *Φ*_f_ and the excitation energy are positively correlated (Figure 2b, d-f), as first noted by Del Vecchio and Blough for NOM,^11^ and by Meredith and Riesz for DOPAm.^54^ A time-domain version of this observation is our finding that lifetimes decrease with increasing excitation wavelength (Table S4). Because there is no reason to expect a collection of molecules to have lifetimes ordered by pump wavelength, these observations point to significant interactions among chromophores. We argue below that a model in which each chromophore is not an isolated molecule but is instead an ultrasmall nanostructure with a tunable transition energy can resolve this conundrum.

The lack of spectral shifting in the hole profile over time indicates that EET between chromophores does not occur within the experimental time window (∼200 fs–3 ns) (Figure S7). The absence of EET is supported by two recent TA studies demonstrating that excited states in synthetic eumelanin are immobile over the same time range and unable to transfer excitation energy to other subunits when excited at visible wavelengths.^40,47^ EWDE similarly reflects restricted EET, as only the chromophores selected by a changing excitation wavelength are capable of emitting.

An important insight comes from the observation that EWDE is most prominent in DOPAm and NOM when excited at visible wavelengths. At sufficiently short wavelengths, the emission maximum becomes independent of excitation wavelength (Figure 4a). Similar behavior has been observed in conjugated polymers at cryogenic temperatures (Figure 4a).^56^ Tuning the excitation pulse across the inhomogeneously broadened band of a conjugated polymer at low temperatures (*T* < 10 K) causes the wavelength of the emission maximum to shift linearly with the excitation wavelength, while maintaining a small Stokes shift until an energy threshold called the ‘localization threshold’ is crossed (Figure 4a).^56^ Above this threshold, EET becomes possible among the coupled units, allowing an excitation formed initially in one segment of the polymer to hop to a lowest energy site (Figure 4b). This energy transfer process causes the emission spectrum to be independent of the excitation energy. As in conjugated polymers, restricted energy transfer has an energy threshold in eumelanin and NOM.

**Figure 4:**
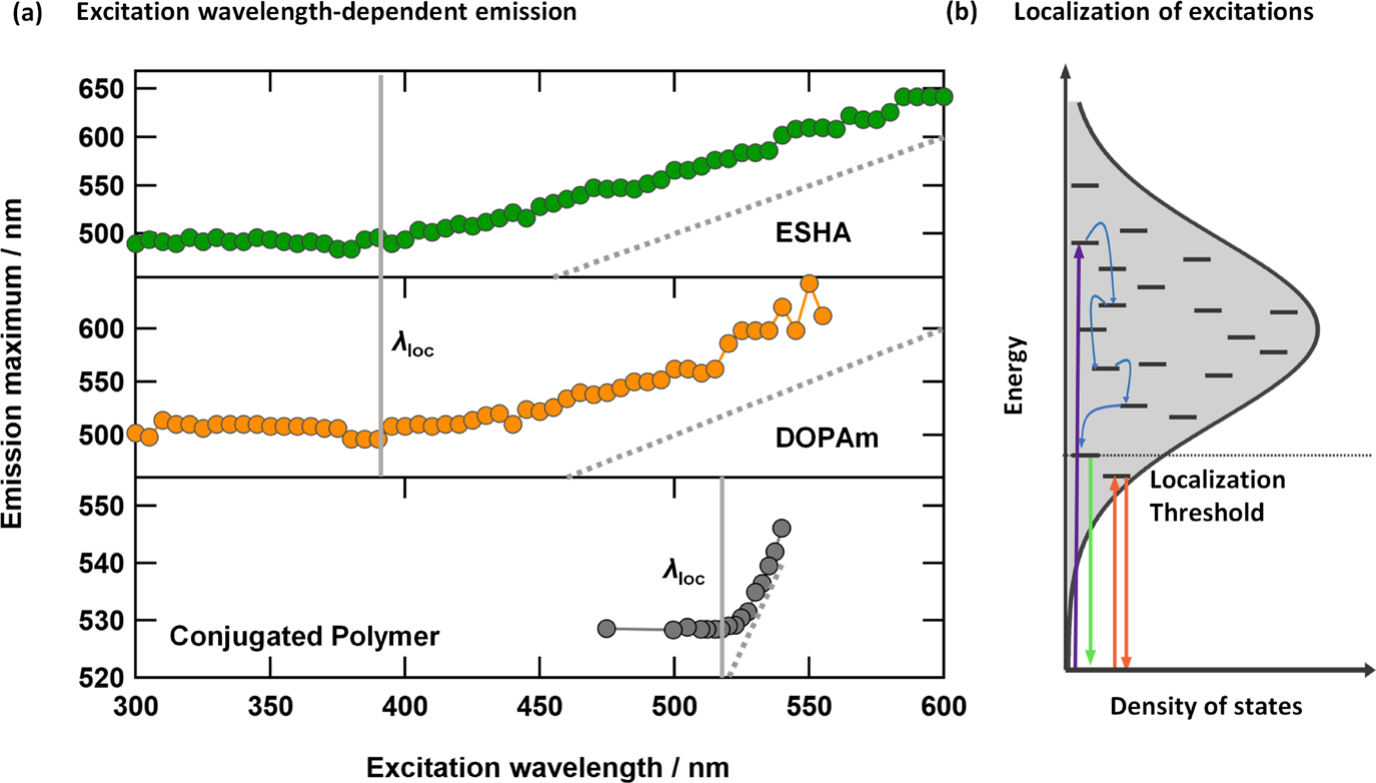
(a) Variation in the intensity-weighted emission maxima of ESHA and DOPAm with excitation wavelength compared with a conjugated polymer film of poly­(p-phenylenevinylene) at 6 K (data from ref 55.) The resonance lines (λ_exc_=λ_em_) are plotted as gray dashed lines. (b) Schematic illustration of EWDE in conjugated polymers. Adapted from ref 56. Copyright 1999 American Chemical Society.

Further support for a localization threshold in NOM comes from subpicosecond time-resolved fluorescence spectroscopy by Yakimov et al., who observed rapid EET in NOM upon near-UV excitation.^57^ In contrast, the transient spectral holes observed in our experiments with visible excitation do not shift spectrally at later times, suggesting that excitations remain localized in this lower-excitation energy regime. Together, these findings support the existence of a threshold below which excitations become localized and do not undergo rapid EET. Most importantly, the observation of a localization threshold in eumelanin and NOM points unambiguously to interchromophoric couplings.

We expect the EET pathways and rates in eumelanin and NOM to be determined by the spatial relationships and couplings between the chromophores much as they are in conjugated polymers. Within a hierarchically structured material like eumelanin, interchromophoric couplings span different length scales, such that some chromophores are strongly coupled while others are not. These length scales depend also on the size of the chromophores. In isolated or very weakly interacting molecules, initial excited states are no larger than the individual molecules, but in supramolecules or disordered solids they could be smaller or larger than the molecular building blocks. For example, in long-chain conjugated polymers, excited states can be localized to short segments containing small numbers of monomers, referred to as “conformational subunits”^48^ or “spectroscopic units”^58^ that are much smaller than full chains due to spatial disorder produced by kinks, bond torsions, and local defects.

In hierarchical materials like melanin and NOM, how large or small the spectroscopic units are compared to structural units will determine how photoproperties evolve when moving between structural levels. If the chromophores or spectroscopic units are much smaller than the structural subunits, then the photoproperties will remain unchanged as more subunits are assembled into higher-order structures. Identifying the point at which the photoproperties first begin to change when disassembling the hierarchical structures found in eumelanin can thus provide an estimate of the chromophore size. This idea is analogous to determining the effective conjugation length of a conjugated polymer by studying the excited-state properties of variable-length oligomers. In the following subsections, we adopt this approach to study the photoproperties of disassembled DOPAm and correlate them with the size of structural units determined by AFM.

### 2.3 Disassembled Eumelanin Subunits Have NOM-like Photoproperties

Proceeding from the hypothesis that the photophysical properties of DOPAm and NOM are tuned by nanostructure, we looked for size-dependent changes in the photoproperties of DOPAm that could identify the size of its chromophores. To accomplish this, a DOPAm sample was disassembled in alkaline solution (Figure 5a) and separated into low molecular weight (LMW) and high molecular weight (HMW) fractions^46^ using a centrifugal concentrator with a 2 kDa nominal molecular weight cutoff (MWCO). Full details are in SI Section S7.

**Figure 5:**
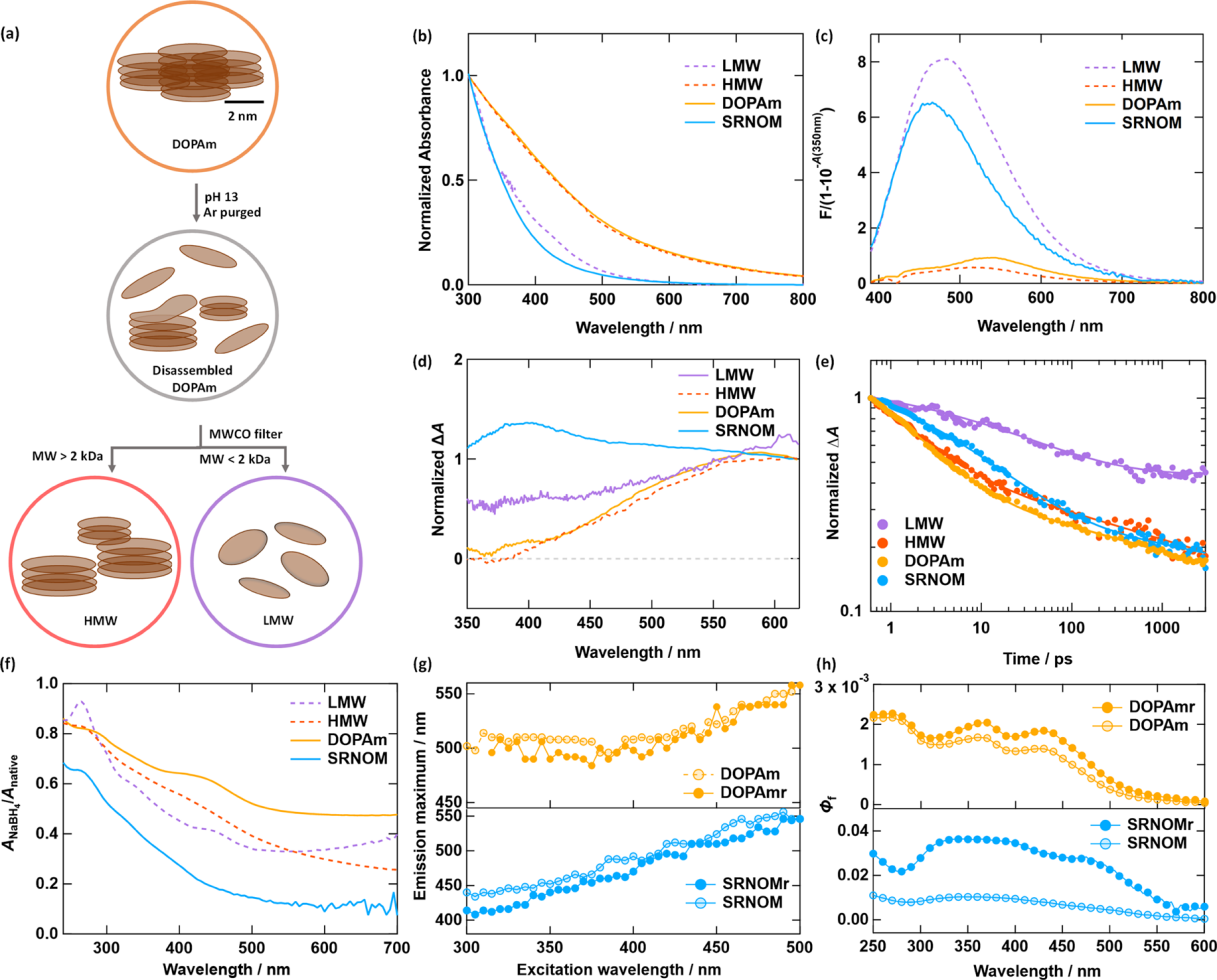
Comparison of steady-state and TA experiments for NOM and disassembled, size-fractionated DOPAm. (a) Alkaline disassembly procedure used to isolate LMW and HMW fractions. (b) Absorption spectra of native DOPAm, disassembled DOPAm (HMW and LMW fractions), and SRNOM normalized at 300 nm. (c) Fluorescence emission spectra divided by the light attenuation, [1–10^–*A*^], excited at 350 nm. (d) TA spectra at 0.5 ps following excitation with 265 nm and normalized at 590 nm. (e) TA kinetic traces for excitation at 265 nm averaged over 590–620 nm and normalized to have unit amplitude at 0.6 ps. (f) Fractional absorbance remaining in the samples after reduction with NaBH_4_. Samples that underwent NaBH_4_ reduction are indicated in the legend by a terminal letter r (e.g., DOPAmr). (g) Blue shift of intensity-weighted emission maxima with reduction in DOPAm and SRNOM is shown in the excitation-dependent emission maxima plots. (h) Increase in fluorescence quantum yield as a function of excitation wavelength with reduction.

The steady-state and transient optical properties of LMW DOPAm are NOM-like, while those of the HMW fraction are nearly indistinguishable from native DOPAm (Figure 5b–e). The absorption spectrum of the LMW fraction of DOPAm is very similar to that of SRNOM (Figure 5b), and the maximum emission wavelength blue shifts compared to native DOPAm and becomes similar to that of SRNOM (Figure 5c). Strikingly, *Φ*_f_ of the LMW DOPAm sample increases more than 10-fold compared to native DOPAm and HMW DOPAm (Figure S9). TA spectra of the LMW fraction excited at 265 nm more closely resemble NOM than native or HMW DOPAm (Figure 5d). In particular, the TA spectrum of the LMW fraction shows greater PIA at short wavelengths than native DOPAm (see Figure S10 for additional delay times). The LMW TA signals also decay significantly more slowly than the native DOPAm signals (Figure 5e, Table S3).

These observations indicate that the photoproperties of DOPAm are sensitive to nanostructure. We propose that the photophysical properties of DOPAm and NOM become more similar as they acquire similar morphologies. To evaluate the extent to which the changes observed postdisassembly arise from altered nanostructure rather than chemical modifications caused by the high pH conditions of disassembly, we explore the effect of chemical modification on the photoproperties in the next subsection.

### 2.4 Borohydride Reduction

To understand the effects of chemical modifications on DOPAm and NOM, we used sodium borohydride (NaBH_4_) reduction to evaluate the role of carbonyl-containing chromophores on the photoproperties of NOM, native DOPAm, and size-fractionated DOPAm, given the known presence of carbonyls in these materials (Figure 1b).

Despite differences in their chemical structures, reduction of NOM and DOPAm (including the HMW and LMW fractions) with NaBH_4_ produces similar changes in optical properties. These changes include decreased absorbance across the UV–visible spectrum, preferential removal of visible absorbance (Figure 5f), blue-shifted emission spectra (Figure 5g, S11a), and enhanced fluorescence quantum yields (Figure 5h, S11b). The preferential removal of visible absorbance (decreasing *A*_NaBH_4__/*A*_native_ with increasing wavelength in Figure 5f) is consistent with prior reports of NaBH_4_ reduction of diverse carbon nanomaterials like NOM,^59^ a water-soluble dihydroxy indole polymer,^26^ atmospheric brown carbon,^60^ carbon dots,^61^ and graphene oxide.^62^ The increase of *Φ*_f_ and the blue-shifted emission after NaBH_4_ reduction have been observed universally in NOM^63^ and more recently in atmospheric brown carbon,^12,64^ highlighting another similarity in the optical behavior of NOM and DOPAm.

These common changes following NaBH_4_ reduction indicate that C=O groups are intimately involved in the optical properties of carbon-based nanomaterials. Because aliphatic and aromatic carbonyl-containing compounds, except for quinones, do not absorb at visible wavelengths unless there is extensive conjugation, the marked decrease in visible absorbance must arise from either the reduction of C=O groups coupled to larger aromatic units or via disruption of interactions between stacked oligomer subunits. While the origin of selective visible absorbance attenuation and enhanced fluorescence is not fully understood, prior work in the NOM and eumelanin fields suggests that interacting chromophore groups (i.e., CT interactions) contribute to low-energy transitions and nonradiative decay pathways, which are suppressed when reduction diminishes these interactions.^12,65^

Although NaBH_4_ reduction produces clear changes in the absorption and emission properties of NOM and DOPAm, these changes are noticeably smaller in magnitude than those observed upon disassembly. For example, the increase in spectral slope from native DOPAm to its LMW fraction is substantially greater than the increase from native to reduced DOPAm (Figure S8). A similar trend is seen in the fluorescence response: With 400 nm excitation, *Φ*_f_ of the LMW fraction is 18× higher than native DOPAm (FigureS9a), whereas the reduced DOPAm sample exhibits only a 1.3× enhancement (Figure 5h). These comparisons demonstrate that the more modest changes induced by NaBH_4_ reduction are insufficient to explain the differences associated with disassembly. This distinction highlights that while chemical structure and redox state influence the photophysics of both systems, they cannot fully account for the pronounced optical differences that emerge when the nanostructure is altered.

### 2.5 Morphological Characterization

Having shown that the DOPAm LMW subunits have NOM-like photoproperties that are produced by morphological and not chemical changes, we sought to characterize the size of the various particles. Past studies employing size exclusion chromatography and field flow fractionation have suggested an upper diameter of ∼1 nm for SRNOM and PPFA.^66,67^ Using an empirical expression that relates the diameter of globular proteins to their molecular weight,^68^ we can crudely estimate that the LMW particles that pass through the 2 kDa MWCO filter have diameters of ***≤*** 2 nm. These sizes are too small to be characterized by DLS (Figure S12d; details in SI Section S9), so AFM was used to image the DOPAm and NOM samples (SI section S10).

AFM images of DOPAm reveal structures with a broad size distribution. The largest structures have lateral dimensions approaching 1 μm with heights between 20–60 nm and appear to be aggregates made of much smaller irregular flakes that are tens of nm across and 1–4 nm in height (Figure 6a,b). Although some aggregation may occur during drying on the mica substrate,^69^ DLS measurements show structures 100 nm–1 μm with a high polydispersity index (0.3) (Figure S12a), indicating that these large aggregates are present in the aqueous dispersions used for spectroscopic measurements.

**Figure 6:**
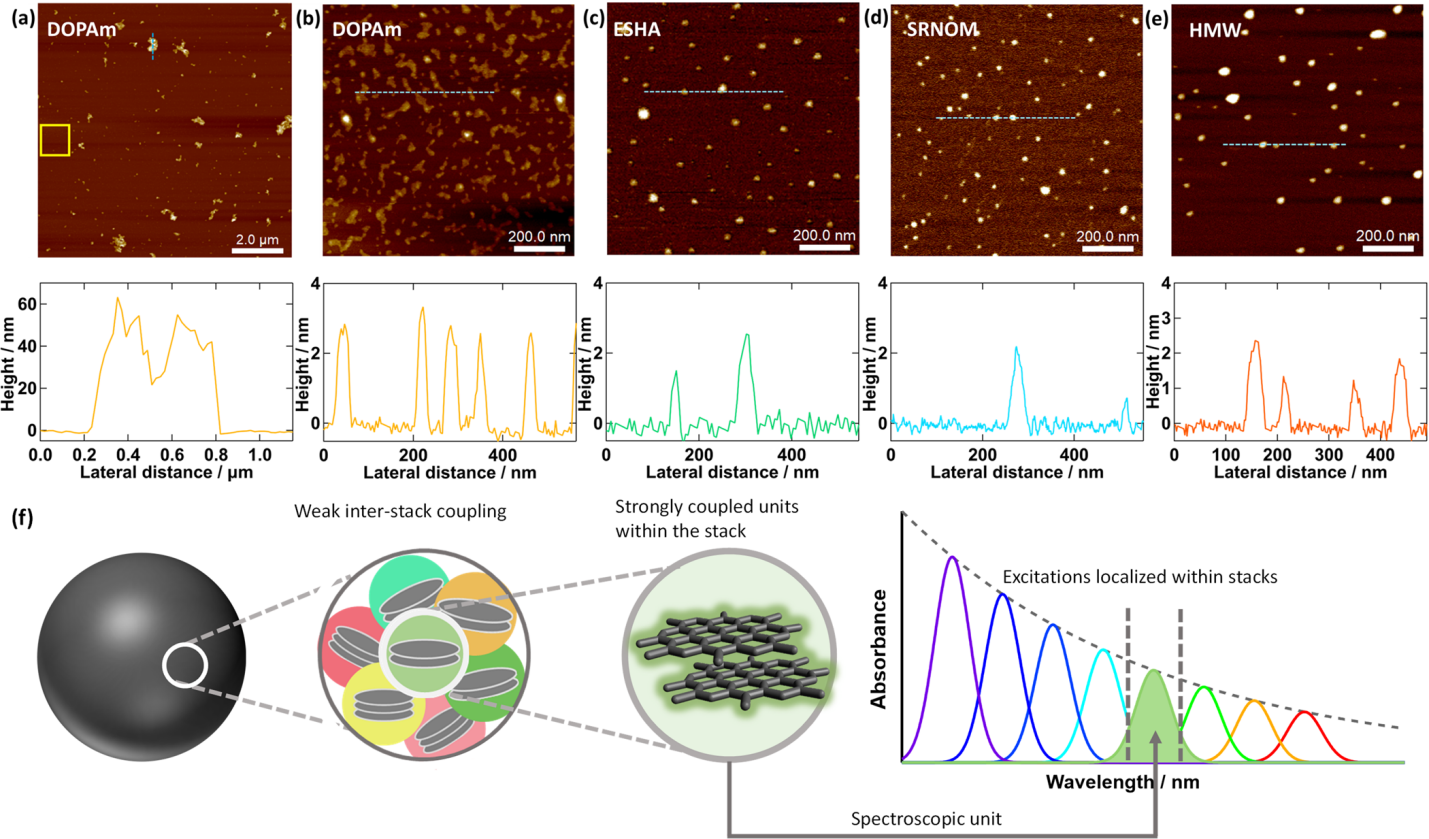
AFM images of (a, b) DOPAm, (c) ESHA, (d) SRNOM, and (e) HMW DOPAm. Panel b is a zoomed-in view of the area in the yellow square in panel (a). The height profile along the blue dashed lines in (a–e) is plotted below the corresponding images. (f) Schematic of the stacked oligomers model illustrating variations in coupling emerging from the hierarchical structure of eumelanin and NOM. The stacks act as the spectroscopic units responsible for visible absorption, with excitations remaining localized within each stack.

AFM images of the NOM samples reveal disk-like morphologies that are uniform in height (1–4 nm) (Figure S13c), consistent with past AFM-imaging studies of NOM.^63,69,70^ Approximately the same range of heights is observed in the smallest DOPAm flakes. While the nanodisks in the NOM samples appear laterally more regular in shape compared to the DOPAm flakes, they still show a range of lateral sizes: The ESHA sample features structures 5–50 nm in diameter, whereas SRNOM and PPFA show slightly smaller disks ranging from 5–25 nm (Figure 6c,d, S14a). Because AFM tip convolution broadens lateral dimensions by ∼4 nm for features 1–3 nm tall,^71^ the smallest observed disks (∼5 nm) likely correspond to true lateral sizes near 1 nm (details in SI Section S10).

The AFM images of the HMW DOPAm sample show nearly circular structures tens of nm in size with consistent heights of 1–4 nm (Figure 6e). Neither the irregular flakes nor the large irregular aggregates observed in native DOPAm are detected. These structures closely match those reported previously for disassembled DOPAm nanoparticles^46^ and are similar to ones observed in the NOM samples in both lateral size and height.

By slightly modifying the disassembly procedure to reduce the concentration of phosphate salts, it was possible to obtain AFM images of LMW DOPAm (Figure S14c; details in Section S10). The LMW particles exhibit heights of 1–4 nm and lateral dimensions ranging from a few nm to 20 nm, comparable to the structures observed in NOM. The morphological changes upon going from native DOPAm to LMW DOPAm may be due to delamination of sheets from small particles, or due to the disruption of H-bonded structures,^46^ but these structural changes are at the limit of the spatial resolution of the AFM instrument used and require further study.

The AFM results show that native DOPAm, HMW, LMW, and NOM samples are dominated by structures with topographical heights consistent with stacks of two or three polycyclic aromatic compounds. Although precisely determining the number of stacked layers by AFM measurements is challenging,^72^ height distributions for the DOPAm and NOM samples extend well above the <1 nm heights measured for single-layer graphene oxide under identical conditions (Figure S13a,b). Prior AFM studies of graphene quantum dots and nanoribbons show 1–2 nm heights for one to three layers.^73,74^ Nanostructures with graphite-like stacking have long been noted in the melanin field,^35,43,47,75^ where they have been described as “stacked sheets”^75^ or “stacked oligomers,”^7^ but they have received less attention in NOM research.^36,63^ These small lamellar structures have a volume on the order of a few nm^3^ and are too small to accommodate a coiled or folded high-MW polymer and too large to accommodate small molecules, supporting the view of NOM and eumelanin as nanoaggregated materials rather than high-molecular weight polymers or macromolecules.^43,76^

The AFM results show that the native DOPAm sample contains a very broad distribution of particle sizes (Figure 6a,b). Although the HMW fraction lacks the largest aggregates of the native sample, it retains the same photoproperties (Figure 5b–e). This indicates that the optical properties are independent of size over the range of sizes present in both samples. The NOM-like photoproperties of the LMW fraction suggest that the photophysical changes only emerge as the DOPAm particles approach the ultrasmall dimensions of the NOM particles. We propose that the spectroscopic units of NOM and eumelanin consist of few-layered stacks of polycyclic aromatic compounds whose size and chemical composition may differ between NOM and eumelanin. In the next subsection, we discuss how restricted energy transfer arises in ensembles of these stacks.

### 2.6 Few-Layer Stacks Explain Restricted EET

We propose that the chromophores governing the common photophysical properties of eumelanin and NOM reside in the smallest stacks seen in the AFM measurements. These stacks are hypothesized to arise from planar, hydrophobic molecules that aggregate readily by *π*-*π* stacking to form few-layered structures consistent with the narrow distribution of heights (1–4 nm) observed in AFM measurements (Figure S13c,d). Hydroxyl, carbonyl, and carboxylate functional groups located on the periphery of molecular units likely also allow lateral aggregation through in-plane hydrogen bonding. The presence of different functional groups will modulate the electronic properties of the molecular units, tuning the transition energies of the CT complexes formed between them.

We propose that individual stacks can support only a sparse set of excited states with only a single excited state in the visible spectrum. This state is likely a low-energy CT state, which is accompanied by higher energy locally excited states that fall outside the visible spectral region. This picture is consistent with single molecule emission experiments on solvothermally synthesized carbon dots, which recently revealed that 3–10 nm particles have just 1–4 chromophores with similar transition energies.^77^ Because each stack only contains a single visible transition, intrastack EET is not possible, explaining the observed EWDE and TSHB (Figure 6f).

Interstack EET is also strongly suppressed within the ensemble of stacks even when the stacks are aggregated into much larger nanoparticles. CT excitations, which have been proposed in eumelanin before,^47,78^ are expected to form electron–hole pairs within a stack that recombine on picosecond time scales. Such rapid recombination competes effectively with energy transfer between stacks separated by several nanometers, where Förster-type EET would occur on time scales of 100 ps to nanoseconds.^79^

Chemical disorder also ensures low rates of EET in aggregated few-layer stacks. Because the longest wavelength transitions are spread across the entire visible spectral region, it is statistically unlikely for chromophores in adjacent stacks to have longest wavelength transitions that are in resonance even when many stacks are aggregated to yield the next level of hierarchical structure. This causes interstack relaxation to no longer be competitive with intraparticle decay pathways, preventing EET under visible excitation. Although this is a tentative and simplistic model, the postulate of an ensemble of few-layer stacks each having a single lowest energy transition that falls somewhere in the visible spectrum explains the restricted energy transfer that gives rise to EWDE and TSHB. The model also explains why the photoproperties of DOPAm are approximately invariant with size above the length scale of a few-layer stack of oligomers.

The absence of EWDE under UV excitation points to a different regime in which energy transfer becomes possible. Ultrafast fluorescence studies of NOM have demonstrated rapid (0.5–1.5 ps) EET upon near-UV excitation, implying energy transfer among chromophores that are separated by <1 nm.^57^ Most stacks possess multiple UV transitions that are close in energy as well as spatially, enabling ultrafast EET. Such dynamics would appear as an ultrafast red shift of the bleach. However, the shortest probe wavelength in our study is 350 nm, and any bleach evolution in the UV lies outside our experimental window. Extending the probe range into UV wavelengths in future studies is needed to directly test this prediction.

The model of chemically disordered few-layer stacks also rationalizes why carbon-based nanomaterials made from chemically distinct planar molecules exhibit similar excited behavior. Universal features can be attributed to the dynamics of CT states (CT excitons) formed between *π*-stacked chromophores. When stacked at an interplanar separation of ∼3.4 Å, different chromophores can form CT excitons with substantial binding energies, which localize the excitation within a single stack. Also, photoexcitation could produce plasmon-like excitations in stacked sheets similar to the ones proposed to occur in PAHs.^80^

A question debated in both the melanin^52,53,54,81^ and NOM^5,12^ fields is whether extended *π* systems are needed to explain long wavelength (i.e., NIR) absorption. Recently, it was demonstrated that a small building block of eumelanin (indole-5,6-quinone) absorbs beyond 900 nm, showing that suitably oxidized small molecules can absorb at surprisingly long wavelengths.^82^ It is intuitive that a large collection of planar molecules that can assemble into few-layer stacks is needed to create a sufficiently diverse set of transitions. This is easily accomplished for NOM, which contains myriad distinct chemical structures derived from diverse biomolecules. Although eumelanin is derived from a single chemical compound, it also exhibits tremendous chemical diversity as over 800 distinct chemical compounds can be generated by joining just two 5,6-dihydroxyindole molecules, the favored eumelanin building blocks.^83^

A further concept that has been emphasized in the NOM field^5,12^ and is seen in our borohydride reduction experiments (Figure 5f), is the importance of CT interactions for generating long-wavelength absorption. This idea has also appeared previously in the melanin field in the form of CT-like transitions from quinhydrone-type complexes (Table 1) and is supported by recent experiments.^47,78^ Together, the ability to pair many chemically distinct stacking units explains how long wavelength absorption can arise in ultrasmall stacks through CT interactions.

## 3 Conclusions and Future Opportunities

We have demonstrated for the first time that the chromophores of both eumelanin and NOM are organized in ultrasmall few-layer stacks only a few nanometers in size made from planar aromatic molecules. This shifts attention to structures that are considerably smaller than the nanostructures typically considered in studies of eumelanin and somewhat larger than the molecular units emphasized in NOM. Although natural melanins are organized on length scales of tens to hundreds of nanometers, our results indicate that their optical properties arise from ensembles of these much smaller stacks. The size-invariant photoproperties explained by our model furthermore account for the preservation of biological functions, such as sunscreening, across hierarchical levels. They also clarify why synthetic nanoparticles like DOPAm, despite containing both ultrasmall stacks and large aggregates, faithfully model the photophysics of natural eumelanin that contains nanoparticles hundreds of nm in diameter.

Our study strongly suggests that tuning or mimicking eumelanin’s photoproperties will require modifying these few-nanometer structures rather than mesoscale aggregates. Although effects due to varying the precise chemical structures still need to be explored, studying a small number of interacting chromophores in an ultrasmall assembly is far more tractable than attempting to engineer larger, hierarchically organized particles. Future work should focus on why these stacks form so readily, the interactions that stabilize them, and their excited-state dynamics.

Because many spectroscopic properties of NOM and eumelanin are ensemble-averaged, deeper insight will require studies of individual stacks or large numbers of stacks with uniform properties. Single-molecule experiments on carbon dots^77,84^ already provide one avenue, while atomically precise model systems such as molecular nanographenes^85,86^ offer another. Study of molecular nanographenes shows that monolayers have structured absorption spectra with strong vibrational progressions, while aggregates containing two or more stacked layers have broader, eumelanin-like absorption spectra,^86^ highlighting how *π*-stacking shapes their absorption spectra. NOM and eumelanin, on the other hand, are likely to be charged because of readily ionizable carboxylate and other functional groups. Because such charges strongly influence *π*–*π* stacking, model systems tailored specifically to eumelanin and NOM are needed.

Our measurements show that NOM and synthetic eumelanin share closely related optical properties but lie along a morphological gradient. DOPAm becomes substantially more NOM-like in optical properties when disassembled into smaller units, underscoring the sensitivity of carbon-based nanomaterials to nanoscale electronic interactions. More work is needed to understand why the photoproperties seen in stacks of NOM and LMW DOPAm differ from stacks of HMW and native DOPAm. Because eumelanin and NOM assemble through both covalent and noncovalent interactions, tracing how photoproperties change across aggregation levels will be necessary to develop a predictive framework.

Beyond photophysics, morphology likely influences other properties such as radical generation and scavenging.^87,88^ Grieco et al. previously showed that TA signals persisting beyond 1 ns are linked to photogenerated radicals in DOPAm.^89^ In analogy to a minimum spectroscopic unit, we wonder if there is a minimum photoactive unit in these materials beyond which radical generation quantum yields become invariant. Future investigations of the relationships between photophysics, radical yields, and morphology are needed to answer this question.

Finally, the resemblance of NOM and DOPAm to other carbon-based nanomaterials (e.g., brown carbon, carbon nanodots, etc.) merits further study, and our work provides a template for future comparisons. Because π-stacking and hydrogen-bonding motifs recur across disordered carbon nanomaterials, the framework developed here for NOM and eumelanin may be valuable for elucidating the photophysics of other carbon-based materials. Understanding how nano- to mesoscale morphology governs the optical properties of eumelanin and NOM opens pathways for designing carbon-based nanomaterials with tailored photophysics, offering promising opportunities in photocatalysis, energy capture and storage, and bioelectronics.

## Supplementary Material





## Data Availability

All data are available in the main text, Supporting Information, and in an Open Science Framework project/data repository for download, free of charge, at https://osf.io/akg69.

## Notes

Experimental details; elemental compositions; additional steady state and TA spectra; TA kinetics and fitting; procedure for isolating transient spectral holes; DLS characterization; additional AFM images and height distributions (PDF)
Transparent Peer Review report available (PDF)

## References

[1] d’Ischia M., Wakamatsu K., Napolitano A., Briganti S., Garcia-Borron J.-C., Kovacs D., Meredith P., Pezzella A., Picardo M., Sarna T., Simon J. D., Ito S. (2013). Melanins and Melanogenesis:
Methods, Standards, Protocols. Pigm. Cell Melanoma
Res..

[2] Motovilov K.
A., Mostert A. B. (2024). Melanin:
Nature’s 4th Bioorganic Polymer. Soft
Matter.

[3] Ghassemi M., Christman R. F. (1968). Properties
of the Yellow Organic Acids of Natural Waters. Limnol. Oceanogr..

[4] Yang X., Rosario-Ortiz F. L., Lei Y., Pan Y., Lei X., Westerhoff P. (2022). Multiple Roles of Dissolved Organic Matter in Advanced
Oxidation Processes. Environ. Sci. Technol..

[5] Korak J. A., McKay G. (2024). Critical Review of
Fluorescence and Absorbance Measurements as Surrogates
for the Molecular Weight and Aromaticity of Dissolved Organic Matter. Environ. Sci. Process. Impacts.

[6] Thurman, E. M. Organic Geochemistry of Natural Waters; Springer, 1985.

[7] Meredith P., Sarna T. (2006). The Physical and Chemical
Properties of Eumelanin. Pigm. Cell Res..

[8] Xie W., Dhinojwala A., Gianneschi N. C., Shawkey M. D. (2024). Interactions of
Melanin with Electromagnetic Radiation: From Fundamentals to Applications. Chem. Rev..

[9] Stenson A. C., Marshall A. G., Cooper W. T. (2003). Exact Masses and
Chemical Formulas
of Individual Suwannee River Fulvic Acids from Ultrahigh Resolution
Electrospray Ionization Fourier Transform Ion Cyclotron Resonance
Mass Spectra. Anal. Chem..

[10] Bahureksa W., Borch T., Young R. B., Weisbrod C. R., Blakney G. T., McKenna A. M. (2022). Improved Dynamic
Range, Resolving Power, and Sensitivity
Achievable with FT-ICR Mass Spectrometry at 21 T Reveals the Hidden
Complexity of Natural Organic Matter. Anal.
Chem..

[11] Del
Vecchio R., Blough N. V. (2004). On the Origin of the Optical Properties
of Humic Substances. Environ. Sci. Technol..

[12] Sharpless C. M., Blough N. V. (2014). The Importance of
Charge-Transfer Interactions in Determining
Chromophoric Dissolved Organic Matter (CDOM) Optical and Photochemical
Properties. Environ. Sci. Process. Impacts.

[13] MacCarthy, P. ; Rice, J. Spectroscopic Methods (Other Than NMR) for Determining Functionality in Humic Substances. In Humic Substances in Soil, Sediment and Water; 1985; pp 527–543.

[14] Chio S.-S., Hyde J. S., Sealy R. C. (1980). Temperature-Dependent
Paramagnetism
in Melanin Polymers. Arch. Biochem. Biophys..

[15] Reuter J. H., Perdue E. M. (1977). Importance of Heavy
Metal-Organic Matter Interactions
in Natural Waters. Geochim. Cosmochim. Acta.

[16] Larsson B., Tjälve H. (1978). Studies on
the Melanin-Affinity of Metal Ions. Acta Physiol.
Scand..

[17] Smit N. P. M., Vink A. A., Kolb R. M., Steenwinkel M.-J. S. T., van den Berg P. T. M., van Nieuwpoort F., Roza L., Pavel S. (2001). Melanin Offers Protection Against
Induction of Cyclobutane Pyrimidine Dimers and 6–4 Photoproducts
by UVB in Cultured Human Melanocytes. Photochem.
Photobiol..

[18] Kvam E., Dahle J. (2003). Pigmented Melanocytes
Are Protected Against Ultraviolet-A-Induced
Membrane Damage. J. Invest. Dermatol..

[19] Blough N. V. (2001). Photochemical
Processes. Encyclopedia of Ocean Sciences.

[20] Steelink C. (1964). Free Radical
Studies of Lignin, Lignin Degradation Products and Soil Humic Acids. Geochim. Cosmochim. Acta.

[21] Sławinski J., Puzyna W., Sławinska D. (1978). Chemiluminescence
in the Photooxidation
of Humic Acids. Photochem. Photobiol..

[22] Sarna T., Dulȩba A., Korytowski W., Swartz H. (1980). Interaction of Melanin
with Oxygen. Arch. Biochem. Biophys..

[23] Senesi N., Miano T. M., Martin J. P. (1987). Elemental,
Functional Infrared and
Free Radical Characterization of Humic Acid-Type Fungal Polymers (Melanins). Biol. Fertil. Soils.

[24] Cataldo F. (1998). On the Structure
of Macromolecules Obtained by Oxidative Polymerization of Polyhydroxyphenols
and Quinones. Polym. Int..

[25] Leresche F., Vialykh E. A., Rosario-Ortiz F. L. (2022). Computational
Calculation of Dissolved
Organic Matter Absorption Spectra. Environ.
Sci. Technol..

[26] Khademimoshgenani N., Green S. A. (2023). Synthesis and Characterization of Humic/Melanin-like
Compounds by Oxidative Polymerization of Simple Aromatic Precursors. Water.

[27] Commoner B., Townsend J., Pake G. E. (1954). Free Radicals
in Biological Materials. Nature.

[28] Rex R. W. (1960). Electron
Paramagnetic Resonance Studies of Stable Free Radicals in Lignins
and Humic Acids. Nature.

[29] Sever R. J., Cope F. W., Polis B. D. (1962). Generation
by Visible Light of Labile
Free Radicals in the Melanin Granules of the Eye. Science.

[30] Senesi N., Schnitzer M. (1977). Effects Of pH, Reaction Time, Chemical Reduction, and
Irradiation on ESR Spectra of Fulvic Acid. Soil
science.

[31] Wilson S. A., Weber J. H. (1977). Electron Spin Resonance Analysis of Semiquinone Free
Radicals of Aquatic and Soil Fulvic and Humic Acids. Anal. Lett..

[32] Nighswander-Rempel S. P., Riesz J., Gilmore J., Meredith P. (2005). A Quantum Yield Map
for Synthetic Eumelanin. J. Chem. Phys..

[33] Felix C. C., Hyde J. S., Sarna T., Sealy R. C. (1978). Interactions of
Melanin with Metal Ions. Electron Spin Resonance Evidence for Chelate
Complexes of Metal Ions with Free Radicals. J. Am. Chem. Soc..

[34] Steelink C., Tollin G. (1962). Stable Free Radicals in Soil Humic Acid. Biochim. Biophys. Acta.

[35] Cheng J., Moss S. C., Eisner M. (1994). X-Ray Characterization
of Melanins—II. Pigm. Cell Res..

[36] Piccolo A. (2001). The Supramolecular
Structure of Humic Substances. Soil Sci..

[37] Chen C.-T., Chuang C., Cao J., Ball V., Ruch D., Buehler M. J. (2014). Excitonic Effects
from Geometric Order and Disorder
Explain Broadband Optical Absorption in Eumelanin. Nat. Commun..

[38] Huijser A., Pezzella A., Sundström V. (2011). Functionality
of Epidermal Melanin
Pigments: Current Knowledge on UV-Dissipative Mechanisms and Research
Perspectives. Phys. Chem. Chem. Phys..

[39] Corani A., Huijser A., Gustavsson T., Markovitsi D., Malmqvist P.-Å., Pezzella A., d’Ischia M., Sundström V. (2014). Superior Photoprotective Motifs and Mechanisms in Eumelanins
Uncovered. J. Am. Chem. Soc..

[40] Ilina A., Thorn K. E., Hume P. A., Wagner I., Tamming R. R., Sutton J. J., Gordon K. C., Andreassend S. K., Chen K., Hodgkiss J. M. (2022). The Photoprotection
Mechanism in
the Black–Brown Pigment Eumelanin. Proc.
Natl. Acad. Sci. U. S. A..

[41] Aiken G. (2014). Fluorescence
and Dissolved Organic Matter: A Chemist’s Perspective. Aquatic Organic Matter Fluorescence.

[42] Buckley S., McKay G., Leresche F., Rosario-Ortiz F. (2024). Inferring
the Molecular Basis for Dissolved Organic Matter Photochemical and
Optical Properties. Environ. Sci. Technol..

[43] Watt A. A. R., Bothma J. P., Meredith P. (2009). The Supramolecular
Structure of Melanin. Soft Matter.

[44] Sun Y.-P., Zhou B., Lin Y., Wang W., Fernando K. A. S., Pathak P., Meziani M. J., Harruff B. A., Wang X., Wang H., Luo P. G., Yang H., Kose M. E., Chen B., Veca L. M., Xie S.-Y. (2006). Quantum-Sized Carbon
Dots for Bright and Colorful Photoluminescence. J. Am. Chem. Soc..

[45] Cayuela A., Soriano M. L., Carrillo-Carrión C., Valcárcel M. (2016). Semiconductor
and Carbon-Based Fluorescent Nanodots: The Need for Consistency. Chem. Commun..

[46] Ju K.-Y., Fischer M. C., Warren W. S. (2018). Understanding the Role of Aggregation
in the Broad Absorption Bands of Eumelanin. ACS Nano.

[47] Kohl F. R., Grieco C., Kohler B. (2020). Ultrafast Spectral Hole Burning Reveals
the Distinct Chromophores in Eumelanin and Their Common Photoresponse. Chem. Sci..

[48] Dykstra T. E., Hennebicq E., Beljonne D., Gierschner J., Claudio G., Bittner E. R., Knoester J., Scholes G. D. (2009). Conformational
Disorder and Ultrafast Exciton Relaxation in PPV-Family Conjugated
Polymers. J. Phys. Chem. B.

[49] Korshin G. V., Li C.-W., Benjamin M. M. (1997). Monitoring
the Properties of Natural
Organic Matter through UV Spectroscopy: A Consistent Theory. Water Res..

[50] Murphy K. R., Stedmon C. A., Graeber D., Bro R. (2013). Fluorescence
Spectroscopy
and Multi-Way Techniques. PARAFAC. Anal. Methods.

[51] McKay G., Korak J. A., Erickson P. R., Latch D. E., McNeill K., Rosario-Ortiz F. L. (2018). The Case
Against Charge Transfer Interactions in Dissolved
Organic Matter Photophysics. Environ. Sci. Technol..

[52] Meredith P., Powell B. J., Riesz J., Nighswander-Rempel S. P., Pederson M. R., Moore E. G. (2006). Towards
Structure–Property–Function
Relationships for Eumelanin. Soft Matter.

[53] Tran M. L., Powell B. J., Meredith P. (2006). Chemical and
Structural Disorder
in Eumelanins: A Possible Explanation for Broadband Absorbance. Biophys. J..

[54] Meredith P., Riesz J. (2004). Radiative Relaxation Quantum Yields
for Synthetic Eumelanin. Photochem. Photobiol..

[55] Heun S., Mahrt R. F., Greiner A., Lemmer U., Bässler H., Halliday D. A., Bradley D. D. C., Burn P. L., Holmes A. B. (1993). Conformational
Effects in Poly­(p-Phenylene Vinylene)­s Revealed by Low-Temperature
Site-Selective Fluorescence. J. Phys.: Condens.
Matter.

[56] Bässler H., Schweitzer B. (1999). Site-Selective
Fluorescence Spectroscopy of Conjugated
Polymers and Oligomers. Acc. Chem. Res..

[57] Yakimov B. P., Rubekina A. A., Budylin G. S., Zherebker A. Y., Kompanets V. O., Chekalin S. V., Vainer Y. G., Fadeev V. V., Gorbunov M. Y., Perminova I. V., Shirshin E. A. (2021). Ultrafast Energy
Transfer Determines the Formation of Fluorescence in DOM and Humic
Substances. Environ. Sci. Technol..

[58] Beenken W. J. D., Pullerits T. (2004). Spectroscopic
Units in Conjugated Polymers: A Quantum
Chemically Founded Concept?. J. Phys. Chem.
B.

[59] Ma J., Del Vecchio R., Golanoski K. S., Boyle E. S., Blough N. V. (2010). Optical
Properties of Humic Substances and CDOM: Effects of Borohydride Reduction. Environ. Sci. Technol..

[60] Phillips S. M., Smith G. D. (2014). Light Absorption by Charge Transfer Complexes in Brown
Carbon Aerosols. Environ. Sci. Technol. Lett..

[61] Zheng H., Wang Q., Long Y., Zhang H., Huang X., Zhu R. (2011). Enhancing the Luminescence
of Carbon Dots with a Reduction Pathway. Chem.
Commun..

[62] Guex L. G., Sacchi B., Peuvot K. F., Andersson R. L., Pourrahimi A. M., Ström V., Farris S., Olsson R. T. (2017). Experimental
Review: Chemical Reduction of Graphene Oxide (GO) to Reduced Graphene
Oxide (rGO) by Aqueous Chemistry. Nanoscale.

[63] Dong Y., Wan L., Cai J., Fang Q., Chi Y., Chen G. (2015). Natural Carbon-Based
Dots from Humic Substances. Sci. Rep..

[64] Phillips S. M., Smith G. D. (2015). Further Evidence
for Charge Transfer Complexes in Brown
Carbon Aerosols from Excitation–Emission Matrix Fluorescence
Spectroscopy. J. Phys. Chem. A.

[65] Mavridi-Printezi A., Menichetti A., Ferrazzano L., Montalti M. (2022). Reversible Supramolecular
Noncovalent Self-Assembly Determines the Optical Properties and the
Formation of Melanin-like Nanoparticles. J.
Phys. Chem. Lett..

[66] Benedetti M. F., Van Riemsdijk W. H., Koopal L. K. (1996). Humic Substances
Considered as a
Heterogeneous Donnan Gel Phase. Environ. Sci.
Technol..

[67] Beckett R., Jue Z., Giddings J. C. (1987). Determination
of molecular weight distributions of
fulvic and humic acids using flow field-flow fractionation. Environ. Sci. Technol..

[68] Erickson H. P. (2009). Size and
Shape of Protein Molecules at the Nanometer Level Determined by Sedimentation,
Gel Filtration, and Electron Microscopy. Biol.
Proced. Online.

[69] Gorham J. M., Wnuk J. D., Shin M., Fairbrother H. (2007). Adsorption
of Natural Organic Matter onto Carbonaceous Surfaces: Atomic Force
Microscopy Study. Environ. Sci. Technol..

[70] Baalousha M., Lead J. R. (2013). Characterization
of Natural and Manufactured Nanoparticles
by Atomic Force Microscopy: Effect of Analysis Mode, Environment and
Sample Preparation. Colloids Surf., A.

[71] Canet-Ferrer J., Coronado E., Forment-Aliaga A., Pinilla-Cienfuegos E. (2014). Correction
of the Tip Convolution Effects in the Imaging of Nanostructures Studied
through Scanning Force Microscopy. Nanotechnology.

[72] Shearer C. J., Slattery A. D., Stapleton A. J., Shapter J. G., Gibson C. T. (2016). Accurate
Thickness Measurement of Graphene. Nanotechnology.

[73] Pan D., Zhang J., Li Z., Wu M. (2010). Hydrothermal Route
for Cutting Graphene Sheets into Blue-Luminescent Graphene Quantum
Dots. Adv. Mater..

[74] Li X., Wang X., Zhang L., Lee S., Dai H. (2008). Chemically
Derived, Ultrasmooth Graphene Nanoribbon Semiconductors. Science.

[75] Zajac G. W., Gallas J. M., Cheng J., Eisner M., Moss S. C., Alvarado-Swaisgood A. E. (1994). The Fundamental Unit of Synthetic
Melanin: A Verification by Tunneling Microscopy of X-Ray Scattering
Results. Biochim. Biophys. Acta BBA - Gen. Subj..

[76] Schaumann G. E. (2006). Soil Organic
Matter beyond Molecular Structure Part I: Macromolecular and Supramolecular
Characteristics. J. Plant Nutr. Soil Sci..

[77] Gomez E., Mehmood A., Bian Z., Lee S. A., Tauzin L. J., Adhikari S., Gruebele M., Levine B. G., Link S. (2025). Single-Particle
Correlated Imaging Reveals Multiple Chromophores in Carbon Dot Fluorescence. J. Am. Chem. Soc..

[78] Petropoulos V., Mavridi-Printezi A., Menichetti A., Mordini D., Kabacinski P., Gianneschi N. C., Montalti M., Maiuri M., Cerullo G. (2024). Sub-50 Fs
Formation of Charge Transfer States Rules the Fate of Photoexcitations
in Eumelanin-Like Materials. J. Phys. Chem.
Lett..

[79] Achermann M., Petruska M. A., Crooker S. A., Klimov V. I. (2003). Picosecond Energy
Transfer in Quantum Dot Langmuir–Blodgett Nanoassemblies. J. Phys. Chem. B.

[80] Lauchner A., Schlather A. E., Manjavacas A., Cui Y., McClain M. J., Stec G. J., García de Abajo F. J., Nordlander P., Halas N. J. (2015). Molecular Plasmonics. Nano Lett..

[81] Pezzella A., Iadonisi A., Valerio S., Panzella L., Napolitano A., Adinolfi M., d’Ischia M. (2009). Disentangling
Eumelanin “Black
Chromophore”: Visible Absorption Changes As Signatures of Oxidation
State- and Aggregation-Dependent Dynamic Interactions in a Model Water-Soluble
5,6-Dihydroxyindole Polymer. J. Am. Chem. Soc..

[82] Wang X., Kinziabulatova L., Bortoli M., Manickoth A., Barilla M. A., Huang H., Blancafort L., Kohler B., Lumb J.-P. (2023). Indole-5,6-Quinones
Display Hallmark
Properties of Eumelanin. Nat. Chem..

[83] Wang J., Blancafort L. (2021). Stability
and Optical Absorption of a Comprehensive
Virtual Library of Minimal Eumelanin Oligomer Models. Angew. Chem., Int. Ed..

[84] Nguyen H. A., Srivastava I., Pan D., Gruebele M. (2021). Ultrafast Nanometric
Imaging of Energy Flow within and between Single Carbon Dots. Proc. Natl. Acad. Sci. U. S. A..

[85] Mahl M., Niyas M. A., Shoyama K., Würthner F. (2022). Multilayer
Stacks of Polycyclic Aromatic Hydrocarbons. Nat. Chem..

[86] Medina-Lopez D., Liu T., Osella S., Levy-Falk H., Rolland N., Elias C., Huber G., Ticku P., Rondin L., Jousselme B., Beljonne D., Lauret J.-S., Campidelli S. (2023). Interplay
of Structure and Photophysics of Individualized Rod-Shaped Graphene
Quantum Dots with up to 132 sp^2^ Carbon Atoms. Nat. Commun..

[87] El
Yakhlifi S., Alfieri M.-L., Arntz Y., Eredia M., Ciesielski A., Samorì P., d’Ischia M., Ball V. (2021). Oxidant-Dependent Antioxidant Activity of Polydopamine Films: The
Chemistry-Morphology Interplay. Colloids Surf.,
A.

[88] Nofsinger J. B., Forest S. E., Eibest L. M., Gold K. A., Simon J. D. (2000). Probing
the Building Blocks of Eumelanins Using Scanning Electron Microscopy. Pigm. Cell Res..

[89] Grieco C., Kohl F. R., Kohler B. (2023). Ultrafast
Radical Photogeneration
Pathways in Eumelanin. Photochem. Photobiol..

