# Supplementary material for: Common Photoproperties of Eumelanin and Natural Organic Matter Emerge from Ensembles of Few-Layered Nanostructures
Source: ACS Cent Sci. 2026 Apr 16;12(5):586–98. doi: 10.1021/acscentsci.5c02304 (PMC13220205; doi:10.1021/acscentsci.5c02304)
Supplement: Supplementary file 1 [file oc5c02304_si_001.pdf]

Supporting Information

Common Photoproperties of Eumelanin and Natural Organic Matter Emerge from Ensembles of Few-Layered Nanostructures

Meera Madhu,[1] Aleksandra Ilina,[1] Hang Li,[2] Garrett McKay,[2,*] and Bern Kohler[1,*]

[1]Department of Chemistry and Biochemistry, The Ohio State University, Columbus, Ohio 43210, United States

[2]Zachry Department of Civil and Environmental Engineering, Texas A&M University, College Station, Texas 77843, United States

Corresponding Authors

Garrett McKay – Zachry Department of Civil and Environmental Engineering, Texas A&M University, College Station, Texas 77843, United States; E-mail: gmckay@tamu.edu

Bern Kohler – Department of Chemistry and Biochemistry, The Ohio State University, Columbus, Ohio 43210, United States; E-mail: kohler@chemistry.ohio-state.edu

Table of Contents

S1 Sample preparation. S3

S2 Chemical composition. S4

S3 ATR-FTIR spectroscopy. S6

S4 UV-Vis-NIR absorption spectroscopy. S6

S5 Steady-state fluorescence spectra. S7

S6 Ultrafast transient absorption spectroscopy. S11

S7 Disassembly of DOPA melanin S20

S8 Sodium borohydride reduction: Steady-state absorption and fluorescence spectra S22

S9 Dynamic light scattering (DLS) measurements. S23

S10 Atomic force microscopy (AFM) measurements S25

S11 References S28

S1. Sample preparation

DOPAm Preparation

DOPAm was synthesized as described previously,[1] following the classic synthesis in which L-DOPA is oxidized by bubbled air in mildly alkaline solution.[2–4] Briefly, 1 g of L-DOPA (Sigma Aldrich, ≥ 98%) was dispersed in 200 mL of ultrapure water in a round bottom flask. The pH of the solution was adjusted to 9.5 by adding ammonium hydroxide (28-30 wt%, Fischer Chemical). Air was bubbled through the continuously stirred reaction mixture for three days. DOPAm was flocculated by the addition of acetonitrile (Sigma Aldrich, ≥ 99.5%). The resulting flocculate was centrifuged at 10,000 g to yield pellets of DOPAm. These pellets were washed 2-3 times with acetonitrile and dried under N_2 for three days, resulting in a black powder of DOPAm that disperses readily in water. While many previous studies that synthesized melanin under basic conditions precipitated DOPAm by lowering the pH of the reaction mixture,[1,2] our procedure uses acetonitrile to precipitate DOPAm, yielding material that is easier to disperse in water.

NOM Sample Descriptions

The NOM samples used in this study are isolate materials obtained from the International Humic Substances Society (IHSS). Our study utilized Suwannee River (SRNOM, 2R101N), Pahokee Peat fulvic acid (PPFA, 1S103F), and Elliott Soil humic acid (ESHA, 5S102H). SRNOM is obtained by reverse osmosis (RO) of water from the Suwannee River (GA, USA), a blackwater river that originates from the Okefenokee Swamp in South Georgia. The Okefenokee has vast peat deposits but most of the dissolved organic carbon is thought to arise from decomposing vegetation. SRNOM is obtained by filtering source water through prefilters to remove particles, cation

exchange resin, and then concentrating the sample in a reverse osmosis system. The RO concentrate is then freeze-dried.

Pahokee peat is an agricultural peat soil of the Florida Everglades, and the IHSS sample was taken from the University of Florida Belle Glade Research Station. Elliot soil is a fertile prairie soil typical of US states like Indiana, Illinois, and Iowa. The IHSS sample was taken from Joliet, Illinois. Humic substances were base extracted from solid soil material with 0.1 M NaOH under a N_2 atmosphere. Humic and fulvic acids are obtained from the supernatant by the operational definition of humic acid insolubility at pH 2.

Safety Statement

No unexpected or unusually high safety hazards were encountered while working with any of the samples. Melanin and NOM materials have low toxicity and high biocompatibility.

S2. Chemical composition

Elemental analyses of DOPAm and the three NOM samples were performed by Hazen Research, Inc. (Golden, CO). The samples were ground and dried in air at 105 ^{o}C to a constant weight before analysis. Dry, ash-free humic acid samples from 12 different soils (non-methylated samples only) have average %C (by mass) of 56.8 $\pm$ 2.5 (σ). %O (by mass) of 34.5 $\pm$ 1.4 (σ), corresponding to an average C:O ratio of 2.2 $\pm$ 0.2 (σ).[5] This agrees with the C:O ratio of 2.2 (Table S1) that we observe for our humic acid sample (ESHA). As summarized in Table S1, the other NOM samples (SRNOM and PPFA) have much lower C:O ratios, indicating a higher degree of oxidation.

The DOPAm sample has a similar C:O ratio as ESHA but is richer in nitrogen, as expected from its indolic subunits. In fact, the DOPAm prepared by us for these studies contains more nitrogen by mass than is typically reported for DOPAm that is prepared by autoxidation. The elemental analysis results from a 1984 study by Sarna and Sealy[6], which are included in Table S1, provide a representative example. The nitrogen enrichment (and lower C:N molar ratio) observed in our DOPAm sample compared to previous studies can be attributed to differences in the procedure used to isolate DOPAm. In our synthesis, acetonitrile is used to precipitate DOPAm from the reaction mixture. This traps ammonium ions derived from the NH_3(aq) used to set the pH during the synthesis in the isolated DOPAm nanoparticles, lowering the C:N ratio. The more common use of precipitating DOPAm from the reaction mixture by adding concentrated HCl(aq) does not trap ammonium ions. We note that evaporating the reaction mixture to isolate DOPAm results in an even lower C:N ratio of 3.7:1, according to an analysis in Cheng et al.[7] These authors did not explicitly state what oxidant was used in their synthesis, but the lower C:N ratio suggests that aqueous ammonia was likely used.

Table S1. Elemental Analysis

	**C % w/w**	**N % w/w**	**H % w/w**	**O % w/w**	**C:N ratio*^b***	**C:O ratio*^b***
**DOPAm**	52.9	12.0	3.2	32.9	5.2	2.1
**DOPAm*^a***	58.0	8.8	2.9	30.4	7.7	2.5
**ESHA**	57.46	4.1	3.7	34.2	16.4	2.2
**SRNOM**	47.44	1.2	4.0	44.4	46.1	1.4
**PPFA**	50.94	2.2	3.4	44.0	27.4	1.5

[a] Results from ref. 6.
[b] Molar ratio.

S3. ATR-FTIR spectroscopy

FTIR absorption spectra were recorded from the same dried powders of DOPAm, SRNOM, PPFA, and ESHA that were used to prepare aqueous dispersions. Spectra were recorded using a Jasco FT/IR 4200 spectrometer equipped with an attenuated total reflection (ATR) PRO450-S accessory with a ZnSe prism. The spectra were recorded by averaging 100 scans from 400-4000 cm^{-1} recorded with 4 cm^{-1} resolution. All spectra were recorded at room temperature under constant purging of the instrument by air from a dry air generator that removes H_2O and CO_2. Baseline correction of the obtained spectra was performed using the Spectra Manager™ Suite Software. Points at 4000 cm^{-1}, 3000 cm^{-1}, and 1800 cm^{-1} were manually selected and connected by straight lines to establish the baseline. The baseline was then subtracted from the spectra to obtain the baseline corrected spectra. Variations in sample penetration depth as a function of frequency and distortions due to anomalous dispersion and frequency-dependent penetration were corrected by applying the ATR correction provided by the manufacturer in the software.

S4. UV-Vis-NIR absorption spectroscopy

DOPAm and NOM solutions for steady-state optical measurements were prepared using 50 mM phosphate buffer (pH 7.0) as the solvent, which facilitated fast and complete dissolution of the solids. All experiments were performed within 48 hours to avoid artifacts due to aging such as slow redox reactions or aggregation. Several independent trials were conducted for most experiments, and the results were reproducible within experimental uncertainty.

UV-visible-NIR spectra of the samples dispersed in 50 mM sodium phosphate buffer at pH 7 were recorded using a Cary 5000 spectrophotometer or Aqualog spectrofluorometer; the latter measures absorbance and fluorescence concomitantly.

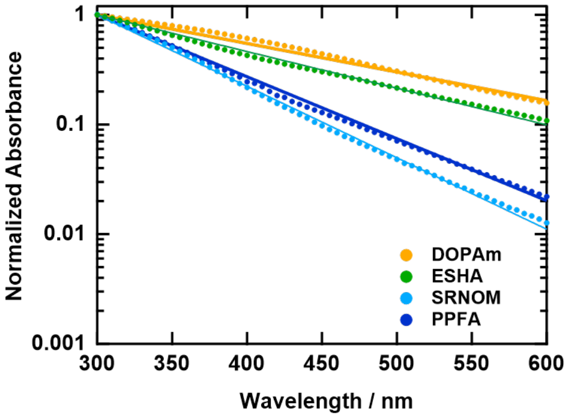

Figure S1. Absorbance spectra of DOPAm and NOM samples normalized at 300 nm (markers) and their fits (solid lines). The spectra were fit to $A_\lambda = e^{-S(\lambda-300)}$. The fit parameters are given in Table S2.

Table S2. Best-fit parameters for the UV/Vis absorption spectra of the samples

Sample	S x 1000 / nm^{-1}
DOPAm	5.58 ± 0.06
ESHA	8.00 ± 0.05
SRNOM	14.65 ± 0.02
PPFA	13.37 ± 0.05

S5. Steady-State fluorescence spectra

Emission spectra were collected between 240 and 800 nm excitation in 10 nm increments using a Horiba Aqualog spectrometer; the emission gain was set to medium with an emission increment (pixels) of 2.33 nm. The integration time was varied depending on the sample's fluorescence efficiency and ranged between 0.1 and 1 s. The Aqualog's excitation monochromator bandpass

is fixed at 4 nm. Fluorescence spectra were processed using standard literature procedures, including blank subtraction, inner filter corrections, and excising Rayleigh scatter. To ensure that inner filter corrections remained effective, the sum of absorbance at the excitation and emission wavelength was kept at less than 1.5 as measured in a 1 cm cell ($A_{\lambda_{\mathrm{ex}}} + A_{\lambda_{\mathrm{em}}} \leq 1.5$).[8] Fluorescence intensities were normalized to the Raman scattering unit area of pure water (measured at a 350 nm excitation wavelength) and were further normalized to the calculated molar concentration of carbon in the samples obtained from the nominal solution concentration and known % m/m of C in the respective samples determined via elemental analysis (Table S1). The emission maximum of the samples, defined here as the intensity-weighted average emission wavelength, calculated as,

$$\lambda_{\mathrm{em}}^{\mathrm{avg}} = \frac{\sum_i F_i \lambda_i}{(\sum_i F_i)}, \quad \text{(S1)}$$

where F_i is the fluorescence intensity measured at wavelength λ_i.

(i) Fluorescence Quantum Yield Calculation

Fluorescence quantum yields (Φ_{f}) were determined according to a previously described method.[9] Briefly, Φ_{f} for NOM and DOPA melanin was determined by comparing the measured fluorescence to that of quinine sulfate,

$$\frac{\Phi_{\mathrm{f,unk}}(\lambda_{ex})}{\Phi_{\mathrm{f,ref}}(350\ \mathrm{nm})} = \frac{n^2}{n_{\mathrm{ref}}^2} \frac{\int_0^\infty I_{\mathrm{unk}}(\lambda_{\mathrm{ex}},\lambda_{\mathrm{em}}) \mathrm{d}\lambda_{\mathrm{em}}}{1-10^{-A_{\mathrm{unk}}(\lambda_{\mathrm{ex}})}} \frac{1-10^{-A_{\mathrm{ref}}(350\ \mathrm{nm})}}{\int_0^\infty I_{\mathrm{ref}}(350\ \mathrm{nm},\lambda_{\mathrm{em}}) \mathrm{d}\lambda_{\mathrm{em}}}, \quad \text{(S2)}$$

where *n* refers to the refractive index, λ_{ex} refers to the excitation wavelength, λ_{em} refers to the emission wavelength, $I(\lambda_{\mathrm{ex}}, \lambda_{\mathrm{em}})$ refers to the fluorescence intensity (in Raman units), $A(\lambda_{\mathrm{ex}})$ refers to the absorbance, and $\Phi_{\mathrm{f,ref}}$ refers to fluorescence quantum yield of quinine sulfate,

which is known to be 0.51 when dissolved in 0.1 N H_2SO_4.[10] The choice of 350 nm excitation for the quinine sulfate reference is due to the fact that quinine sulfate does not absorb at wavelengths > 400 nm, which are excitation wavelengths of interest for NOM and DOPAm. This approach is justified by the fact that instrument-specific correction factors were used to correct raw fluorescence data $I(\lambda_{\mathrm{ex}}, \lambda_{\mathrm{em}})$. Further, the difference in refractive indices of the unknown and reference solution was deemed to be negligible ($n^2/n_{\mathrm{ref}}^2 \cong 1$). Integrals were performed using the trapezoidal method in MATLAB (trapz) after setting $I(\lambda_{\mathrm{ex}}, \lambda_{\mathrm{em}}) < 0$ (due to instrumental noise) to 0 to ensure no negative contributions to the area.

The absolute values and excitation wavelength-dependence of Φ_{f} yields for NOM and DOPAm (Figure 2b, S2a) measured in our study were generally in agreement with past literature. For example, the measured Φ_{f} at 370 nm excitation for PPFA and SRNOM of 0.0131 and 0.0102. These values are within 30% and <1% of values reported for PPFA (0.0108) and SRNOM (0.0100) at 370 nm excitation reported previously.[9] The Φ_{f} values measured in our study are approximately two-fold larger than those reported previously by Meredith and co-workers,[11] although it is important to note that there are some differences in the melanin samples. In ref. 11, tyrosine (4-hydroxyphenylalanine) was polymerized whereas in our study dopamine (3,4-dihydroxyphenylalanine) was the starting material. In addition, the ref. 11 study measured fluorescence at pH 10 (to help solubilize) melanin, while in our study solution pH was buffered at 7 with 50 mM phosphate.

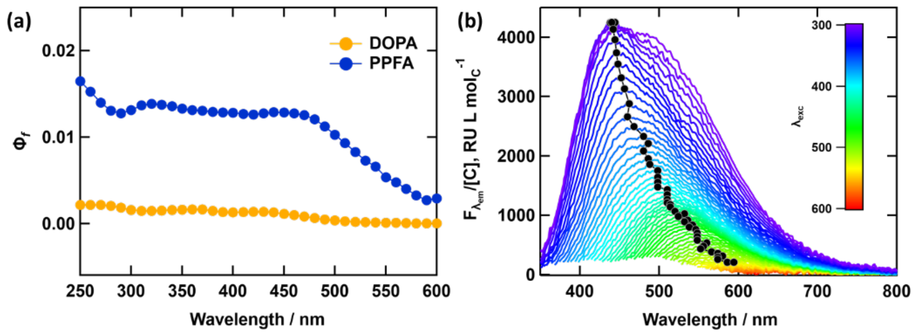

Figure S2. (a) Fluorescence quantum yields of PPFA as a function of excitation wavelength. The trace for DOPAm is reproduced from Figure 2 for comparison. (b) Emission spectra of PPFA at different excitation wavelengths. The color map represents increasing wavelength of excitation with dark blue for 300 nm excitation and red for 600 nm. The black data markers represent the maximum emission wavelength for each emission spectra.

S6. Ultrafast transient absorption spectroscopy

Femtosecond broadband TA measurements were performed using a previously described spectrometer.[12] The spectrum of the pump pulse shown in Figure 3 was measured using a mini spectrometer (Ocean Optics Flame) with a fiber optic sampling. A half-wave plate was utilized to change the angle between the linearly polarized pump and probe pulses. Pump fluences are reported as on-axis (r = 0) values for pulses assumed to have Gaussian profiles in space and time. The probe beam was focused to a spot size of 200 μm at the sample. A liquid flow cell, consisting of a 1 mm thick PTFE spacer sandwiched between two CaF_2 windows of the thickness of 1 mm (front) and 2 mm (back), was used to circulate about 4 mL solutions of the samples. A gradient neutral density filter was used to attenuate the pump at each wavelength. Native DOPAm, NOM and disassembled melanin samples were measured at 9×10^{15} cm^{-3} excitation density. Fresh 4 mL aliquots diluted from a 4.37 mg mL^{-1} and 2.91 mg mL^{-1} stock solutions of SRNOM and PPFA, respectively, were used for collecting data at each excitation wavelength. Fresh 4 mL solutions of native DOPAm were prepared for each measurement. The HMW fraction of disassembled DOPAm was diluted from the collected HMW fraction while LMW fraction was used without dilution. The absorbances of the samples at different wavelengths are PPFA- 0.39 (500 nm), 0.41 (400 nm), 0.52 (265 nm); SRNOM-0.23 (500 nm), 0.39 (400 nm), 0.50(265 nm); native DOPAm - 0.14 (500 nm), 0.27 (400 nm) 0.50 (265 nm); LMW-0.17 (265 nm); HMW-0.19 (265 nm). No changes were observed in the UV-Vis absorption spectrum of the samples after the transient absorption experiments.

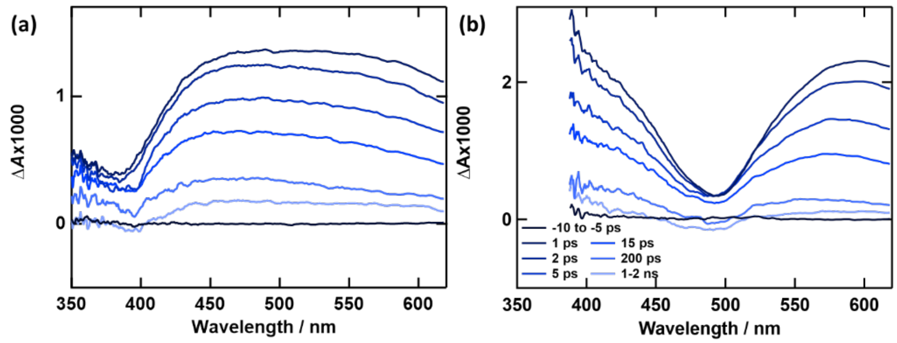

Figure S3. Transient absorption spectra (measured at the delay times shown in the legend in b) of PPFA recorded with excitation wavelengths of (a) 400 nm and (b) 500 nm.

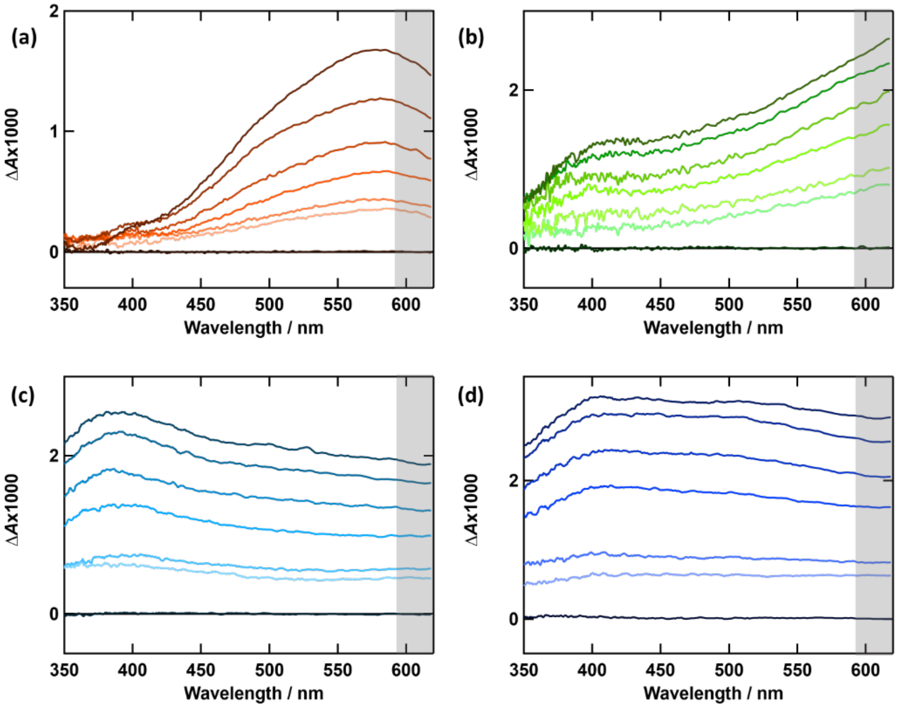

Figure S4. Transient absorption spectra recorded with excitation wavelengths of 265 nm of (a) DOPAm, (b) ESHA, (c) SRNOM and (d) PPFA spectra (measured at the delay times shown in the legends in Figure 3d-f and S3b, respectively).

(i) Excited state decay kinetics and fits

The DOPAm and NOM kinetic traces were fit to a model used previously to describe the TA kinetics of DOPAm,[33]

$$\Delta A(t) = A_1(\lambda_{\mathrm{ex}})e^{-\left(\frac{t}{\tau}\right)^{\beta}} + A_2(\lambda_{\mathrm{ex}})t^{-\alpha} \quad \text{(S3)}$$

In eq. S3, the first term describes a stretched exponential function with time constant τ and stretching parameter, β, while the second term describes a power law decay with exponent α. Parameters A_1 and A_2 are amplitudes that vary with the excitation wavelength, λ_{ex}. At sufficiently long delay times the stretched exponential term in eq. S3 decays to zero and the long-time decay is dominated by the power law term. The DOPAm and NOM signals decay at long delay times according to a power law as seen from the straight-line character of the long-time signals when they are graphed on a log-log plot (Figure S5b). Eq. S3 yielded satisfactory fits to the DOPAm and NOM kinetic traces as revealed by plots of the fit residuals (Figure S6). The time constants for the stretched exponential are about one order of magnitude smaller for DOPAm (~0.2 ps) relative to the NOM (> 1 ps) samples, matching the trend in experimental half-lives (Table S3).

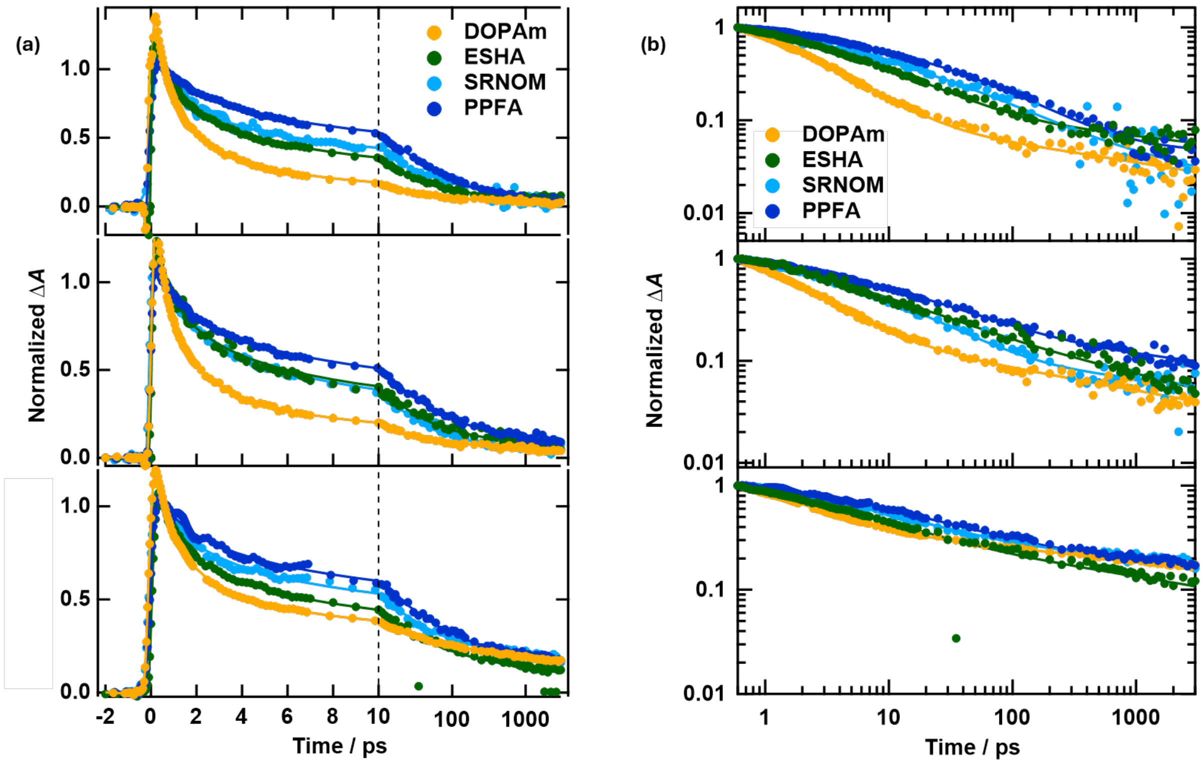

Figure S5. (a) Excited state decay kinetics of DOPAm, ESHA, SRNOM, and PPFA observed for 500 nm, 400 nm and 265 nm excitation. The kinetic traces, which were scaled to have the same amplitude at 0.6 ps, were obtained by averaging the measured TA signals over probe wavelengths from 590-620 nm (2.1-2.0 eV), corresponding to the gray bars in Figure 3 and S4. The horizontal (time) axis is logarithmic after 10 ps (vertical dashed line) to better visualize the slowest decay components. (b) The same datasets are shown on a log-log plot.

Table S3. Best-fit parameters to kinetic traces[a]

**Sample**	$\boldsymbol{\lambda_{\mathrm{exc}}}$ **(nm)**	$\boldsymbol{A_1}$	$\boldsymbol{A_2}$	$\boldsymbol{\alpha}$	$\boldsymbol{\tau}$ **(ps)**	$\boldsymbol{t_{1/2}}$ **(ps)**
DOPAm	265	2.51 ± 0.14	0.442 ± 0.022	0.12 ± 0.09	0.16 ± 0.03	3.3
	400	3.9 ± 0.3	0.25 ± 0.04	0.23± 0.03	0.120 ± 0.022	1.6
	500	3.96 ± 0.22	0.17 ± 0.05	0.24 ± 0.06	0.150 ± 0.024	1.2
DOPAm, HMW	265	1.10 ± 0.20	0.52 ± 0.03	0.127 ± 0.010	0.16 ± 0.04	3.8
DOPAm, LMW	265	0.87 ± 0.14	0.37 ± 0.12	0.021 ± 0.012	15.6 ± 2.2	200
ESHA	265	0.95 ± 0.20	0.56 ± 0.08	0.19 ± 0.03	0.79 ± 0.22	5.6
	400	1.3 ± 0.8	0.46 ± 0.14	0.24 ± 0.10	0.9 ± 0.4	3.6
	500	1.76 ± 0.20	0.24 ± 0.08	0.18 ± 0.06	0.91 ± 0.14	2.9
PPFA	265	0.94 ± 0.16	0.45 ± 0.08	0.12 ± 0.03	4.4 ± 0.6	17
	400	1.01 ± 0.12	0.40 ± 0.06	0.179± 0.024	3.3 ± 0.3	8.9
	500	1.24 ± 0.08	0.19 ± 0.04	0.19 ± 0.03	7.7 ± 0.6	12
SRNOM	265	1.14 ± 0.10	0.42 ± 0.06	0.102 ± 0.020	1.8± 0.3	11
	400	1.65 ± 0.16	0.25 ± 0.06	0.19 ± 0.04	1.23 ± 0.12	4.7
	500	0.77 ± 0.20	0.42 ± 0.08	0.32 ± 0.04	5.1 ± 1.1	4.5

[a]TA data from Figure S5, except for the disassembled samples (DOPAm HMW and LMW), which are from Figure 5e. The kinetic traces were recorded at the indicated excitation wavelengths (λ_{ex}) and averaged over probe wavelengths between 590-620 nm. The averaged kinetic traces were then scaled to have unit amplitude at 0.6 ps before fitting to eq. S3. Only data points in the range 0.6-1000 ps were included in the fit with β fixed at 0.33 (1/3), while all remaining parameters were optimized. The half-life value ($t_{1/2}$) is the delay time when the fitting function equals 0.5. All uncertainties in the Table are 2σ values.

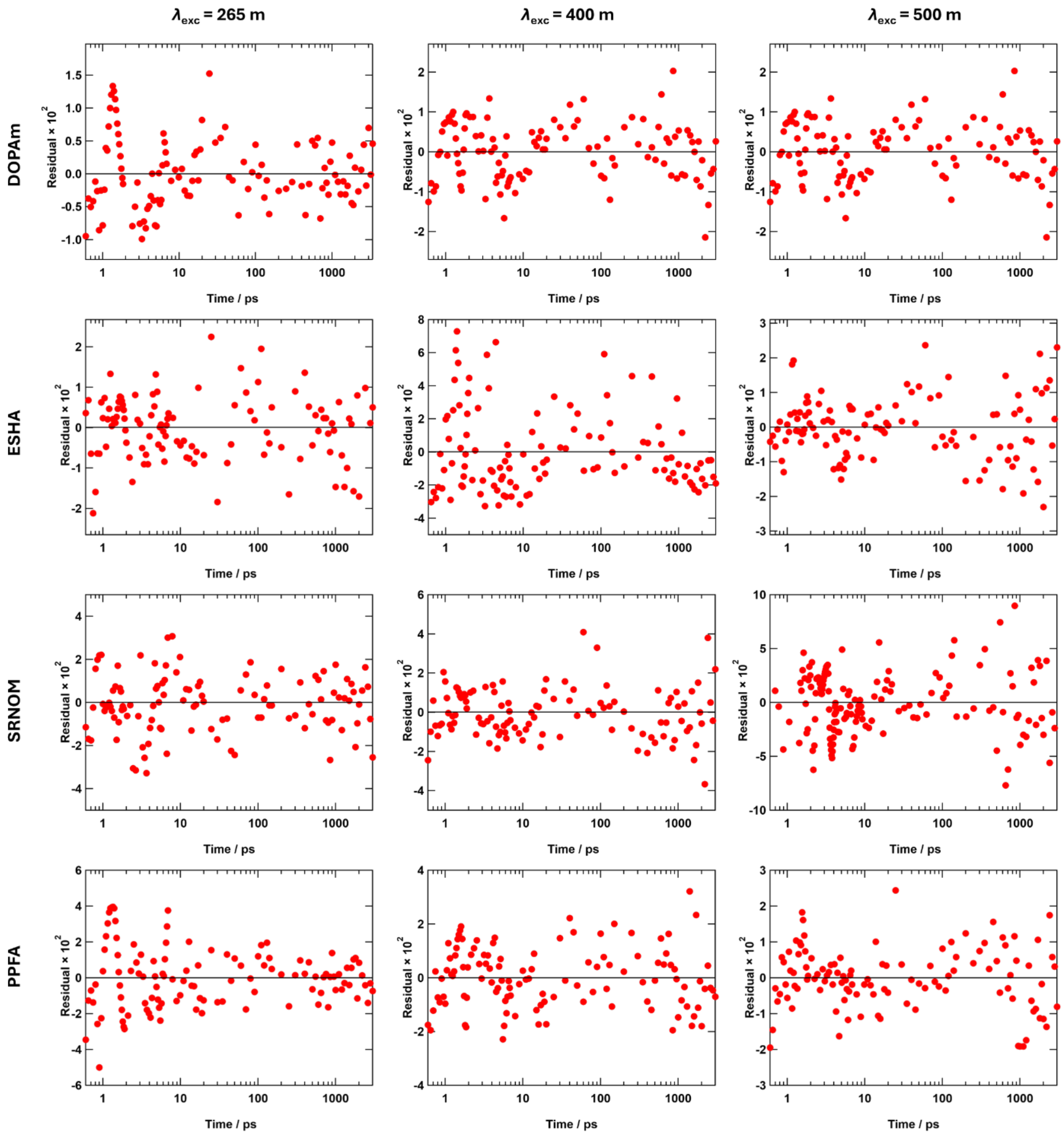

Figure S6. Fit residuals from fits to DOPAm, ESHA, SRNOM, and PPFA transient absorption kinetic traces with excitation at 265 nm, 400 nm, and 500 nm. The residuals are plotted against time to assess the quality of the fit of the transient absorption spectra data. The residuals are scattered randomly around zero without any discernible pattern, indicating a good fit of the stretched exponential-power law model to the data.

(ii) Procedure used to isolate the spectral hole profile

The fs-TA signals in Figure 3 showing TSHB record an absorbance difference (ΔA) and can therefore have positive and negative contributions. Positive signals arise from photoinduced absorption (PA), while negative ones are due to ground-state bleaching (GSB) or stimulated emission (SE). A dip or hole is observed in the transient spectrum when positive PA contributions over a broad range of probe wavelengths are reduced by negative signal contributions that occur in a narrower spectral window that is approximately centered about the pump wavelength. As seen in Figure 3, the negative signal contributions sometimes dominate, causing the sign of the total signal to be negative, while in other cases, they merely reduce the magnitude of a positive signal. SE is ruled out for the DOPAm and NOM samples because the transient holes are nearly centered about the excitation wavelength. A transient spectral hole caused by SE would be centered at considerably longer wavelengths given the Stokes shifts of up to 100 nm in these samples (Figure 2d-f). The negative-going signals in Figure 3 are thus assigned to bleaching of the ground state population by the excitation pulse when it is tuned to visible wavelengths.

The spectral holes were isolated from the transient absorption spectra at 400 nm and 500 nm excitation following the previously reported procedure.[1] At each time delay, the transient absorption spectrum with 265 nm excitation consisting of photoinduced absorption (PIA) was scaled with a factor of *s* to match the signal around 620 nm (2.0 eV), where bleaching is not expected. The scaled PIA was subtracted from the transient absorption spectra with the visible excitations according to the following equation,

$$\Delta A^{\mathrm{GSB}}\left(\upsilon_{\mathrm{pu}}^{\mathrm{vis}}, \upsilon_{\mathrm{pr}}\right) = \Delta A\left(\upsilon_{\mathrm{pu}}^{\mathrm{vis}}, \upsilon_{\mathrm{pr}}\right) - s\Delta A\left(\upsilon_{\mathrm{pu}}^{\mathrm{UV}}, \upsilon_{\mathrm{pr}}\right), \quad \text{(S4)}$$

where υ_{pu} and υ_{pr} are the pump and probe frequencies, respectively.

The spectral holes obtained were fit to a negative Gaussian function of the form,

$$G(E,t) = -a(t)e^{-0.5\left\{\frac{E-E_0(t)}{\frac{\mathrm{FWHM}(t)}{2.355}}\right\}^2}, \tag{S5}$$

where E is the probe energy, $a(t)$ is a positive scaling factor that depends on the pump-probe delay time, t, $E_0(t)$ is the peak center and $\mathrm{FWHM}(t)$ is the full width at half maximum of the isolated spectral hole.

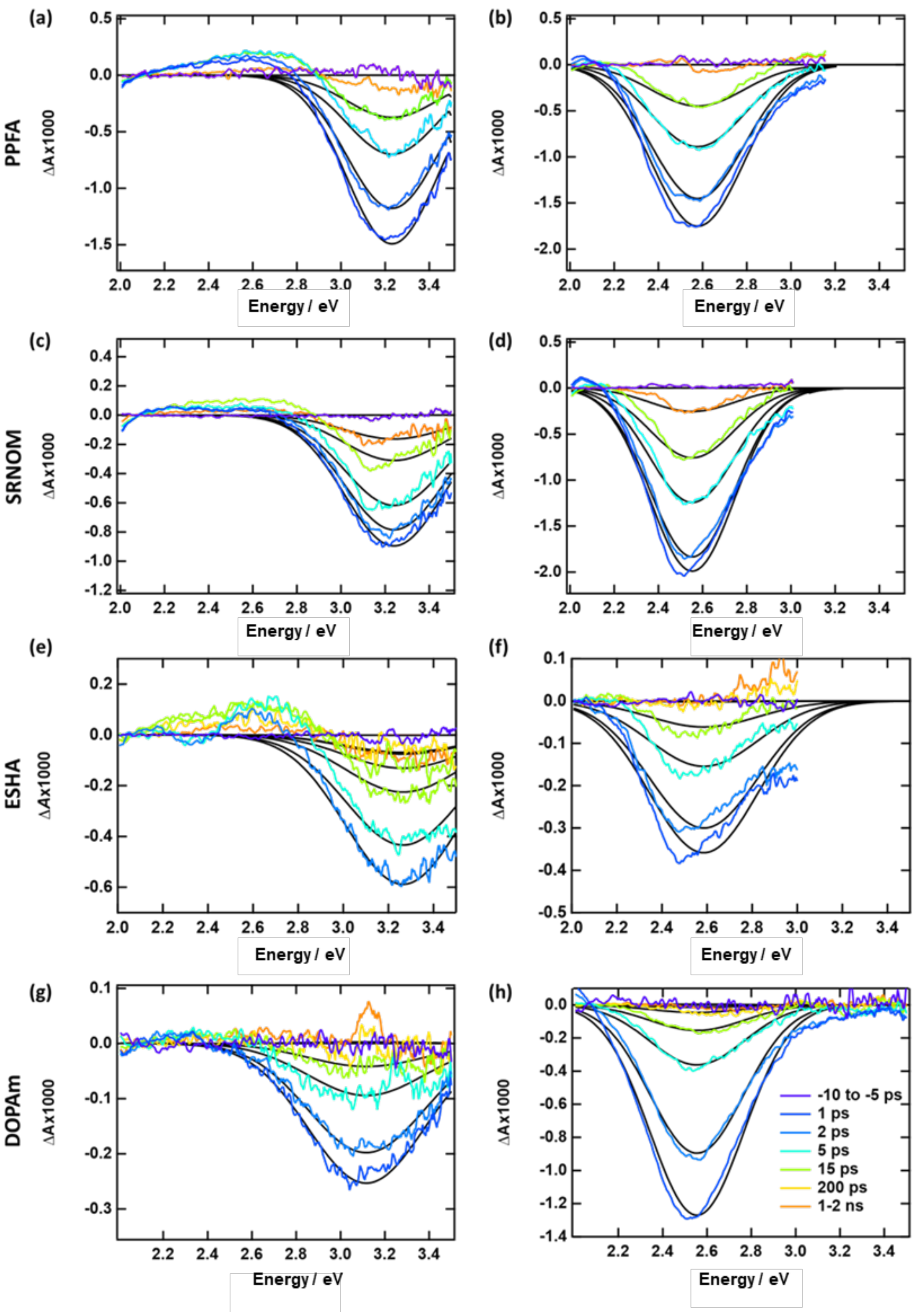

Figure S7. Holes extracted from transient absorption experiments with excitation at 400 nm (left column) and 500 nm (right column) on PPFA (a, b), SRNOM (c, d), ESHA (e, f), and DOPAm (g, h).

Table S4. Hole width (eV, FWHM) at 1 ps from spectral hole burning with excitation at 400 nm or 500 nm.[a]

Sample	400 nm (3.10 eV)		500 nm (2.48 eV)	
	E_0	FWHM	E_0	FWHM
PPFA	3.21 ± 0.02	0.48 ± 0.05	2.56 ± 0.06	0.49 ± 0.05
SRNOM	3.22 ± 0.02	0.53 ± 0.05	2.58 ± 0.06	0.45 ± 0.04
ESHA	3.22 ± 0.02	0.60 ± 0.06	2.58 ± 0.06	0.57 ± 0.06
DOPAm	3.15± 0.02	0.47 ± 0.05	2.55 ± 0.05	0.60 ± 0.06

[a]The reported uncertainties are 2σ.

S7. Disassembly of DOPA melanin

The pH-controlled disassembly of DOPAm was carried out by adapting the procedure reported by Ju et al.[13] Briefly, 10 mL of 1 mg mL^{-1} of DOPAm in ultrapure water was purged with argon for 30 minutes to remove dissolved air. Next, 1 mL of 1 M NaOH, which had also been purged with argon, was added to raise the pH to ~13. After 5 h, the solution was neutralized by adding 2 mL of an argon-purged solution of 1 M KH_2PO_4 to halt disassembly. The resulting solution was centrifuged in a Vivaspin® 15R centrifugal concentrator with a 2 kDa cut-off for 60 minutes at 6,000 ×g. Following the recommendation of the manufacturer, the concentrator was pre-rinsed with ultrapure water before use to remove trace amounts of glycerol or sodium azide left over from manufacturing. The low molecular weight fraction (LMW) with MW < 2000 Da was obtained by collecting the fraction that passed through the membrane, while the high molecular weight fraction (HMW) with MW > 2000 Da was obtained by collecting the fraction remaining in the concentrator pocket.

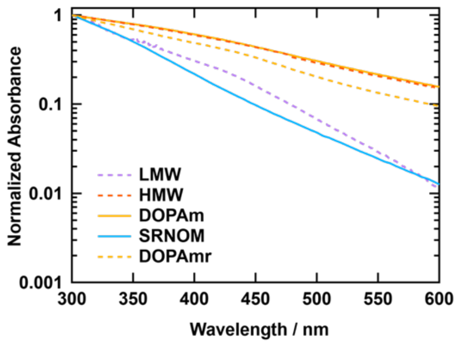

Figure S8. Semi-log plot of the absorbance spectra of native and disassembled DOPAm along with SRNOM normalized at 300 nm. The absorbance spectrum of reduced DOPAm is included for comparison with disassembled DOPAm samples.

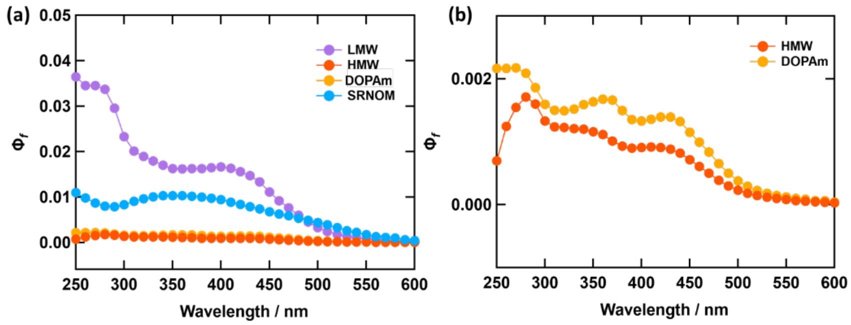

Figure S9. (a) Fluorescence quantum yields, Φ_f, of DOPAm and NOM samples measured at different excitation wavelengths. (b) Φ_f of DOPAm and HMW plotted on a magnified scale.

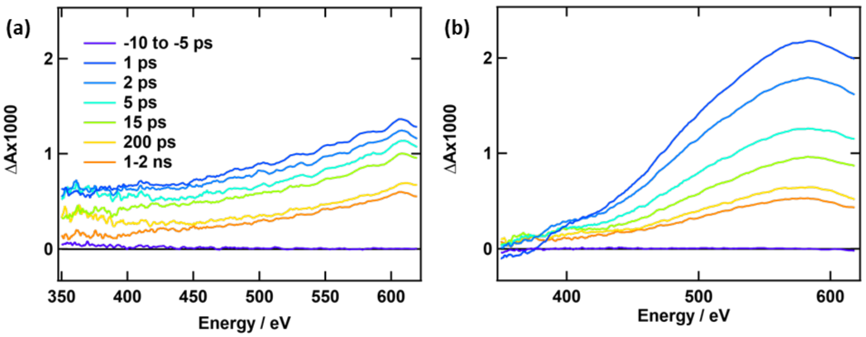

Figure S10. Transient absorption spectra measured with 265 nm excitation of (a) LMW fraction and (b) HMW fraction at the delay times shown in the legend in panel a.

S8. Sodium borohydride reduction: Steady-state absorption and fluorescence spectra

Sodium borohydride ($NaBH_4$) reductions were performed under aerobic conditions. A concentrated stock solution (100 mg/mL) of $NaBH_4$ was prepared by adding 300 mg of solid slowly to 3 mL of pH 12 water followed by stirring for ~1 hr. Because the molar concentration of borohydride-reducible groups in DOPAm and NOM is unknown, $NaBH_4$ was added at a 25-fold mass excess with respect to the molar carbon concentration (determined via solution concentration and % m/m carbon of the solid). Prior work has shown that pH 10 is reached within the first few minutes after adding $NaBH_4$ to an unbuffered solution.[14] Because the NOM and DOPAm dispersions were prepared in the presence of a pH buffer, the pH of each sample was adjusted to ~10 prior to $NaBH_4$ addition. Reduction reactions were quenched after 24 h by adjusting the pH to 7. Pre- and post-reduction spectra were measured at pH 7 to facilitate comparison. Given the high buffering capacity of the solution (50 mM phosphate), these pH adjustments necessitated accounting for dilution in the measured optical spectra (volume change < 10%). For reduction experiments on of disassembled DOPAm, 25-fold mass excess of NaBH4 was added to a 0.15 mg/mL dispersion prepared by diluting HMW fraction obtained post-disassembly and to the as-obtained LMW fraction (0.2 mg/mL). The concentrations of fractions obtained following disassembly were estimated by subtracting the mass concentration determined by drying a known volume of the HMW fraction from the mass concentration of the DOPAm used.

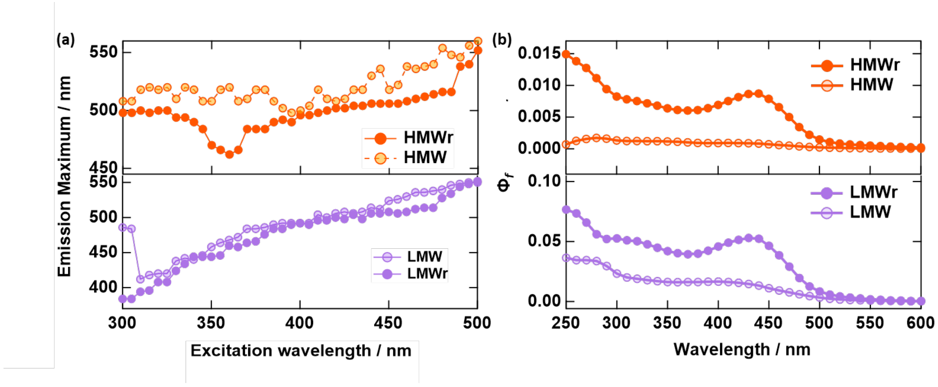

Figure S11. (a) Blue shift of emission maxima shown in the excitation-dependent emission maxima plots with reduction in HMW and LMW. (b) Increase in quantum yield as a function of excitation wavelength with reduction.

S9. Dynamic light scattering (DLS) measurements

DLS measurements were performed using a Malvern Panalytical Zetasizer Pro instrument equipped with a He-Ne laser (633 nm). The measurements were taken at a scattering angle of 173°. For these measurements, 0.1 mg/mL dispersions of the samples were prepared by diluting freshly prepared 3 mg/mL stock solutions in pH 7 sodium phosphate buffer. Each 1 mL aliquot of the 0.1 mg/mL dispersions was measured in a 1 cm path length fused silica cuvette. Prior to measurement, the dispersions were filtered through a 1.0 µm PTFE syringe filter to eliminate any dust particles. Data acquisition and analysis were performed using Malvern Zetasizer Nano software. For each sample, three consecutive DLS measurements were taken, each with an acquisition time of 20 seconds. The average hydrodynamic radius is reported along with the standard deviation calculated from the three measurements as provided by the software.

The DLS measurement is expressed as an intensity-weighted size distribution. This distribution can be converted into a volume-weighted distribution using the refractive index to understand the relative concentrations of different-sized particles in the samples. Based on

previous reports of the refractive index for DOPAm and NOM, a value of 1.55 was used for all samples. The conversion from intensity-weighted to number-weighted distribution was performed using the Malvern Zetasizer Nano Software.

For SRNOM and PPFA samples, the measured scattering light intensity was insufficient to obtain a reliable size distribution. This indicates that these samples have very few particles large enough (> a few nm) to be detected. Higher concentrations could not be used as the high absorbance and fluorescence of the samples interfered with the scattered light.

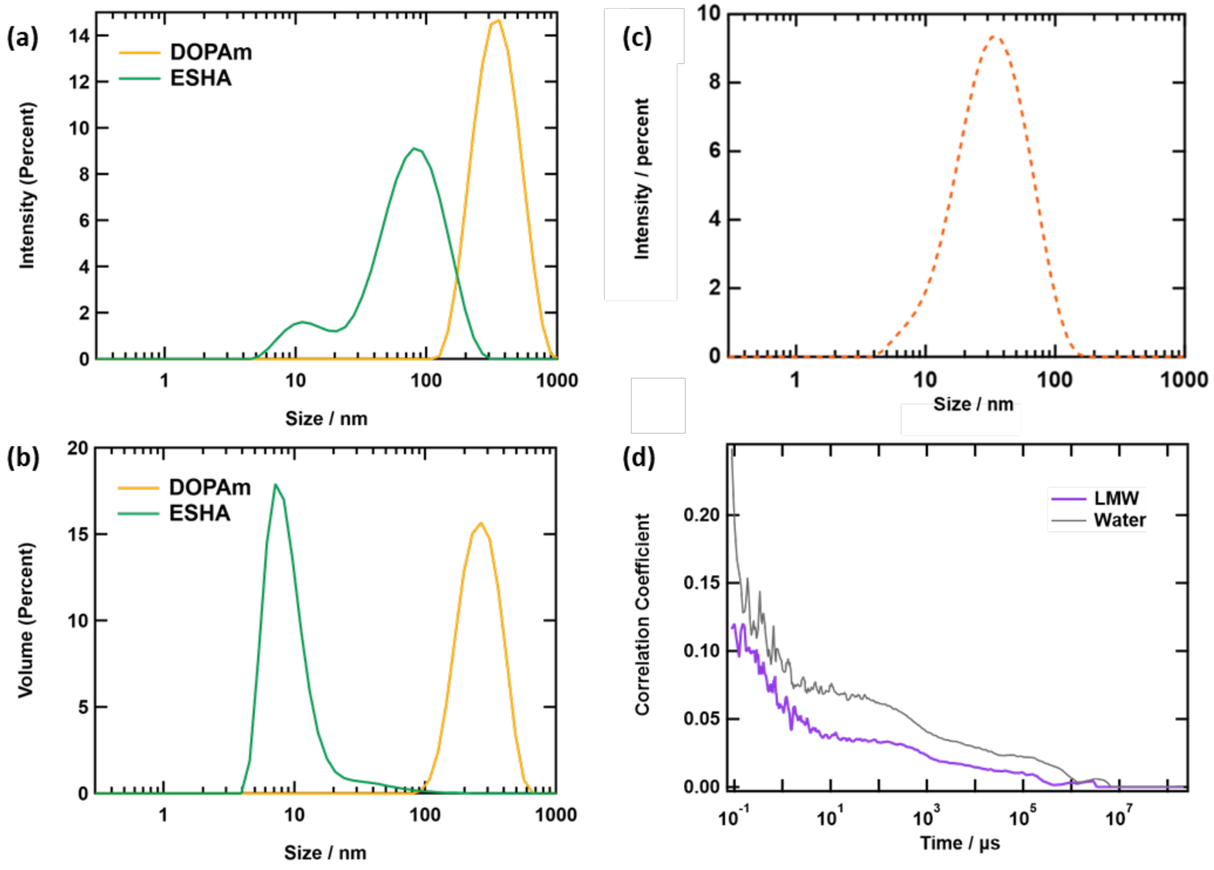

Figure S12. (a) Intensity size distribution of DOPAm and ESHA obtained from DLS measurements. The average size of DOPAm particles is 320 nm. ESHA exhibits two populations centered around 14 nm and 92 nm. (b) Volume distribution, derived from the measured intensity distribution in a. (c) Size distribution of HMW fraction of DOPAm. The average diameter of HMW particles is 37 nm in size. (d) The autocorrelation function of LMW and water.

S10. Atomic force microscopy (AFM) measurements

AFM measurements were performed using a Bruker Dimension Icon instrument operated in ScanAsyst™ mode, an off-resonance tapping mode that automatically optimizes imaging parameters during scanning. Images were collected under ambient conditions at a scan size of 1 µm × 1 µm, resolution of 512 × 512 pixels, and scan rate of 0.99 Hz using Bruker ScanAsyst-Air probes (nominal tip radius 2 nm, resonance frequency 45-95 kHz, spring constant 0.2–0.8 N m^{-1}). Stock dispersions of each sample were prepared in deionized water at a concentration of 2 mg mL^{-1}. The phosphate buffer that was used to prepare dispersions for other experiments was not used to minimize the concentration of salts that can enhance particle aggregation on the mica substrate.[15] To make it easier to disperse in water, the pH of the ESHA sample was adjusted to 10 using 1 M NaOH. LMW DOPAm samples were prepared by a modified disassembly procedure, in which the pH of the DOPAm dispersion after 5 h of alkaline disassembly was neutralized with 1 M HCl. All other steps of the disassembly protocol were unchanged. The resulting LMW fraction was then dialyzed for 24 h using a 0.5–1 kDa cellulose ester membrane (Spectra/Por 131057) to remove residual salts from the disassembly process. Single-layer graphene oxide powder (ACS Material, LLC) was used as received. It was dispersed in water by sonicating for 30 minutes to prepare a 0.2 mg mL^{-1} stock solution.

For AFM imaging, aliquots of the freshly prepared stocks were diluted to 30 µg mL^{-1}, and 50 µL of the resulting dispersion was dropcast onto freshly cleaved mica substrates. The pH of all dropcast solutions were measured to be around 7. The samples were then dried under nitrogen for 30 minutes prior to imaging. Lateral broadening due to tip convolution effects were estimated

using the procedure of Canet-Ferrer et al.[16] For a nominal tip radius of 2 nm, the lateral broadening for structures around 1-2 nm was estimated to be about 4 nm.

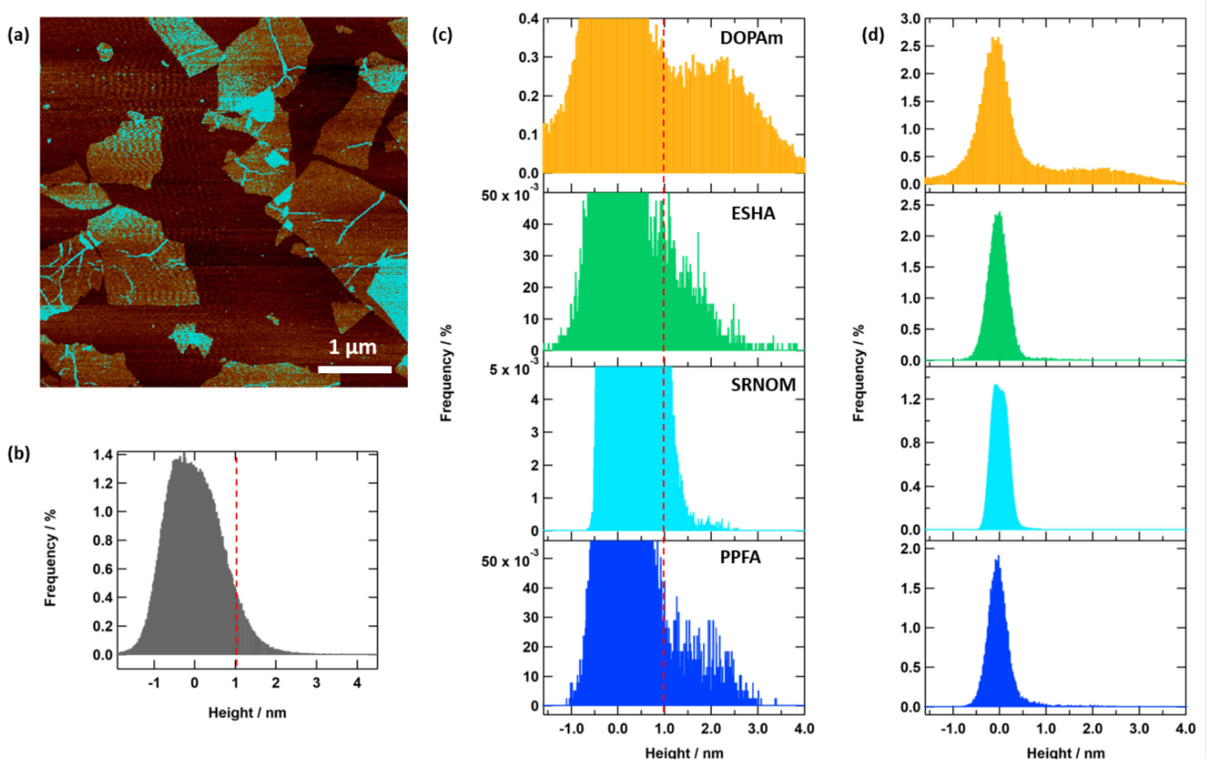

Figure S13. (a) AFM image of single-layer graphene oxide sheets. (b) Histogram showing the height distribution of every pixel in panel a. The number of bins was set to 512. Features that are above 1 nm (marked by the red dashed line) are marked in cyan in panel a. These areas correspond to either defects on the single layers or regions with multiple layers. (c) Histograms of height distributions from the AFM images of the samples in Figure 6a,c,d and PPFA. A magnified view along the *y*-axis is plotted to highlight the heights corresponding to the particles. (d) The complete view of the height distribution of the samples. The prominent peaks around zero correspond to the bare mica background.

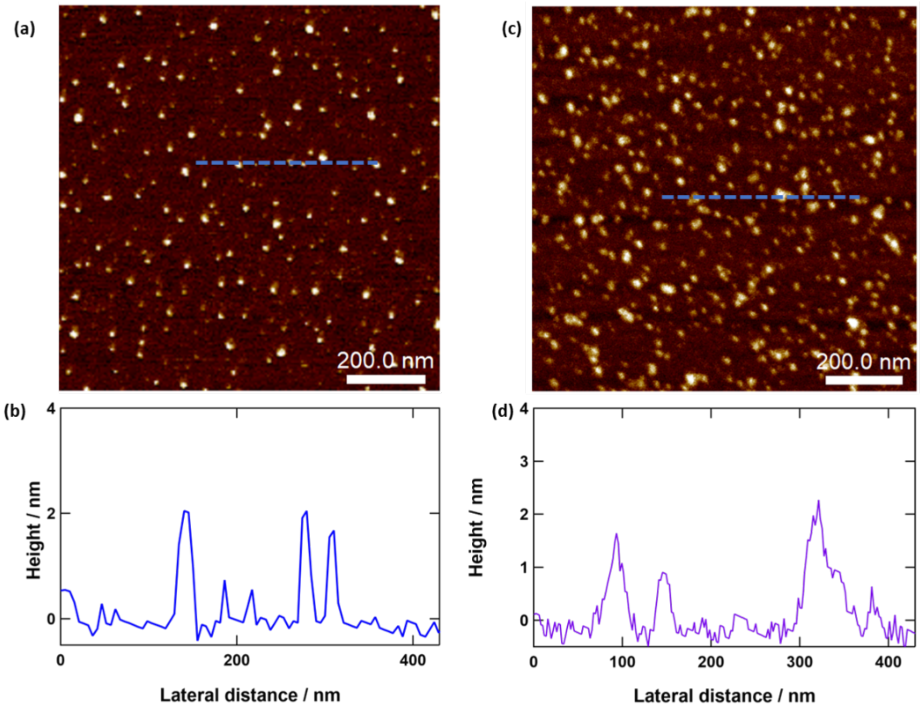

Figure S14. AFM image of (a) PPFA and (b) the height profile along the blue dashed line in panel a. (c) LMW fraction and (d) the height profile along the blue dashed line in panel c.

S11. References

(1) Kohl, F. R.; Grieco, C.; Kohler, B. Ultrafast Spectral Hole Burning Reveals the Distinct Chromophores in Eumelanin and Their Common Photoresponse. *Chem. Sci.* **2020**, *11*, 1248–1259.

(2) Arnow, L. E. The Preparation of Dopa-Melanin. *Science* **1938**, *87*, 308–308.

(3) Binns, F.; King, J. A. G.; Mishra, S. N.; Percival, A.; Robson, N. C.; Swan, G. A. Studies Related to the Chemistry of Melanins. Part XIII. Studies on the Structure of Dopamine-Melanin. *J. Chem. Soc.* **1970**, 2063-2070.

(4) Felix, C. C.; Hyde, J. S.; Sarna, T.; Sealy, R. C. Interactions of Melanin with Metal Ions. Electron Spin Resonance Evidence for Chelate Complexes of Metal Ions with Free Radicals. *J. Am. Chem. Soc.* **1978**, *100*, 3922–3926.

(5) Riffaldi, R.; Schnitzer, M. Electron Spin Resonance Spectrometry of Humic Substances. *Soil Sci. Soc. Am. J.* **1972**, *36*, 301–305.

(6) Sarna, T.; Sealy, R. C. Photoinduced Oxygen Consumption in Melanin Systems. Action Spectra and Quantum Yields for Eumelanin and Synthetic Melanin. *Photochem. Photobiol.* **1984**, *39*, 69–74.

(7) Cheng, J.; Moss, S. C.; Eisner, M.; Zschack, P. X-Ray Characterization of Melanins—I. *Pigm. Cell Res.* **1994**, *7*, 255–262.

(8) Kothawala, D. N.; Murphy, K. R.; Stedmon, C. A.; Weyhenmeyer, G. A.; Tranvik, L. J. Inner Filter Correction of Dissolved Organic Matter Fluorescence. *Limnol. Oceanogr.:Methods* **2013**, *11*, 616–630.

(9) McKay, G.; Korak, J. A.; Erickson, P. R.; Latch, D. E.; McNeill, K.; Rosario-Ortiz, F. L. The Case Against Charge Transfer Interactions in Dissolved Organic Matter Photophysics. *Environ. Sci. Technol.* **2018**, *52*, 406–414.

(10) Velapoldi, R. A.; Mielenz, K. D. *Standard Reference Materials: A Fluorescence Standard Reference Material*; National Institute of Standards and Technology, 1980.

(11) Nighswander-Rempel, S. P.; Riesz, J.; Gilmore, J.; Meredith, P. A Quantum Yield Map for Synthetic Eumelanin. *J. Chem. Phys.* **2005**, *123*, 194901.

(12) Grieco, C.; Kohl, F. R.; Zhang, Y.; Natarajan, S.; Blancafort, L.; Kohler, B. Intermolecular Hydrogen Bonding Modulates O-H Photodissociation in Molecular Aggregates of a Catechol Derivative. *Photochem. Photobiol.* **2019**, *95*, 163–175.

(13) Ju, K.-Y.; Kang, J.; Chang, J. H.; Lee, J.-K. Clue to Understanding the Janus Behavior of Eumelanin: Investigating the Relationship between Hierarchical Assembly Structure of Eumelanin and Its Photophysical Properties. *Biomacromolecules* **2016**, *17*, 2860–2872.

(14) Schendorf, T. M.; Del Vecchio, R.; Koech, K.; Blough, N. V. A Standard Protocol for $NaBH_4$ Reduction of CDOM and HS. *Limnol. Oceanogr.:Methods* **2016**, *14*, 414–423.

(15) Baalousha, M.; Lead, J. R. Characterization of Natural and Manufactured Nanoparticles by Atomic Force Microscopy: Effect of Analysis Mode, Environment and Sample Preparation. *Colloids Surf., A* **2013**, *419*, 238–247.

(16) Canet-Ferrer, J.; Coronado, E.; Forment-Aliaga, A.; Pinilla-Cienfuegos, E. Correction of the Tip Convolution Effects in the Imaging of Nanostructures Studied through Scanning Force Microscopy. *Nanotechnology* **2014**, *25*, 395703.
