# Supplementary material for: Common Photoproperties of Eumelanin and Natural Organic Matter Emerge from Ensembles of Few-Layered Nanostructures
Source: ACS Cent Sci. 2026 Apr 16;12(5):586–98. doi: 10.1021/acscentsci.5c02304 (PMC13220205; doi:10.1021/acscentsci.5c02304)
Supplement: Supplementary file 2 [file oc5c02304_si_002.pdf]

oc-2025-02304h.R1

Name: Peer Review Information for "Common Photoproperties of Eumelanin and Natural Organic Matter Emerge from Ensembles of Few-Layered Nanostructures"

First Round of Reviewer Comments

Reviewer: 1

Comments to the Author

In their manuscript entitled “Common Photoproperties of Eumelanin and Natural Organic Matter Emerge from Ensembles of Few-Layered Nanostructures”, McKay, Kohler and coworkers report how basic photoproperties that include transient spectral hole burning and excitation-wavelength dependent emission arise in melanin and natural organic matter from the ensemble behaviour of these ultrasmall units. From the measurements presented in this manuscript, the authors propose that NOM and synthetic melanin share closely related optical properties but lie along a morphological gradient. DOPAm becomes substantially more NOM-like when disassembled into smaller units, emphasizing the sensitivity of carbon-rich nanomaterials to electronic interactions. Overall, the manuscript presents very high-quality measurements to support the claims made that are central to the understanding of the optical properties of eumelanin. The manuscript is recommended for publication in the present form.

Reviewer: 2

Comments to the Author

Many photophysical studies of melanin and related natural materials have been reported. I suspect even more have been attempted and abandoned. The problem is that the absorption is broad and unstructured, making it difficult to pinpoint what species are absorbing the light. This paper established a foundational viewpoint for future studies. Not only does the work provide an appealing testable model for eumelanin photophysics, but it

established a unified framework for comparable materials. The paper focuses in that regard on natural organic matter, but the work will also inspire new carbon dot research.

I had several questions as I read the paper, but it turned out that they were nicely addressed in page 25 and beyond. The importance of aggregates appeared to be incompatible with the observed lack of energy transfer, but that was addressed. I like the disorder density of states model, Fig 4, from Heinz Bässler, it is a key advance of the work to base the model around this picture. Indeed, that same picture proved important for understanding disorder in conjugated polymers. I was also wondering about CT states, but, again, this is addressed. A related important comment in the work is that substitution of these small molecules can have a profound influence on the photophysics.

Overall, I think this paper has potential to be highly influential. It should be published.

Reviewer: 3

Comments to the Author

The authors use spectroscopy to demonstrate similarity at the nanoscale leading to similar photophysical properties between synthetic melanin and natural organic matter. The data are convincing, the manuscript is well-written and clear, and the implications for future research are interesting. My only suggestion is that the authors include some measurement of natural melanin, or an explanation of why use of synthetic melanin is justified here, given that the point of the paper is to compare two natural forms of melanin. If they argue that structure of synthetic melanin is close enough to natural melanin for use here, they should clearly explain why.

P5L3: I would not attribute a “functional role” to NOM.. this implies (as does “allow”) that it has evolved to perform this role when of course it is non-living material that does not evolve. “their similar properties cause melanin pigments and nom to have similar effects” might be a better way to phrase it

P8L17: Since the paper is framed as a comparison between the mechanisms of natural melanin and NOM, it seems less than optimal to use synthetic melanin. Why not use sepia or some other natural melanin source? I

Reviewer: 4

Comments to the Author

The manuscript presents an interesting study focussed on apparent similarities between melanin and derivatives, and “molecular organic matter” which is a broad definition of multiple compounds and mixtures thereof arising from decomposition of organic matter.

The authors hint at the potential for unified formalism to explain properties of all nanomaterials. This would be very important.

The authors’ main point is “parallel photophysics of melanin and NOM.”

It is difficult to understand what is meant by “parallel photophysics”, but from the context of the article this statement may refer to the similarities of photophysical properties which in the authors’ view originate from similar morphologies.

The last sentence of the introduction states that “Taken together, this work advances understanding of how the optical properties are tuned by particle morphology and identifies length scales at which key optical properties emerge.”

This sentence can be applied to a very broad range of materials and is not specific. The authors use AFM, steady-state absorption and emission spectroscopy, and time-resolved spectroscopy to investigate the properties of two groups of substances.

It is stated that “NOM and melanin exhibit remarkably similar steady-state and transient photo-properties, with some second order differences. “ It is not clear what “second-order differences” refer to. Further, the data presented show the similarities which appear to be purely coincidental.

There are multiple materials (for example, molecular organic nanosheets) which would exhibit the same properties.

Whilst the manuscript raises interesting questions about serendipity in Nature, and as such may be of interest to the broad readership of any journal, the scientific arguments presented in the paper do not support the physico-chemical claims of the similarities of the properties. It is difficult to follow what the key argument is – is this that when molecules pack together in nanostructures (the authors use the term "ultrasmall" without quantifying it), they have similar optical properties? This can not be universally correct. The evidence presented does not support the claim of fundamental similarities, but rather coincidental properties. Further, the work on the analysis of steady-state spectra raises serious concerns (see below).

Thus, despite a list of interesting observations, it is not possible to support publication of this study. The ultrafast spectroscopy part is of high quality and could be potentially published separately.

Detailed comments are given below.

1. "The electronic absorption spectra of both DOPAm and the NOM samples are broad, featureless, and decrease monotonically from UV to NIR wavelengths (Figure 2a). The spectra decay exponentially with wavelength such that a graph of log absorbance vs. wavelength yields an approximately straight line."

There are multiple (potentially unlimited) number of molecules and materials that exhibit a long "tail" absorption across visible range and into NIR. This is usually attributed to a number of individual electronic transitions, at slightly different energies due to slightly different environments. This is typical for aggregates of light-absorbing molecules and is often observed when aggregates of multiple compositions are formed (e.g. dimer, trimers, tetramers etc existing in the same solution). Unless there is a specific physical (chemical) reason for the stated log dependence, it can also originate from scattering. The log dependence appears overinterpreted, and a careful examination of Fig S1 indicate that the dependence is not linear. Any dependence of the absorbance should relate to the energy of the transition not the wavelength (unless this is a discussion of quantum confinement). It is possible that – as in carbon-based nanomaterials – there is a distribution of band-gaps causing the continuous absorption feature. But this in itself does not mean that melanin and the decomposition products are similar in properties. (In perhaps a crude analogy, there are multiple compounds that absorb around 400 nm. This does not imply that their properties are similar or related).

2. Fig 2 FTIR – the full spectrum is presented, which has some groups of interest identified. These are not labelled, and whilst they are likely be (CO) groups, amide groups, and -CH, this figure needs to be much clearer.

3. Figure 2d,e,f is unclear. The black dots represent some shift of the emission maximum? What does the white spectrum underneath the emission spectra represent? The dependence of QY (Fig 2b) on the excitation wavelength requires further analysis. The absorption spectra presented are normalised. Thus it is not clear which optical density was used to record each emission spectrum The authors state (just above Fig S1 in the SI) that they corrected for inner-filter effect. They also state the optical densities for emission measurements were up to 1.5. It is therefore not possible to agree that the measurements have been done correctly.

The authors describe that "Fluorescence intensities were normalized to the Raman scattering unit area (measured at a 350 nm excitation wavelength) and were further normalized to the calculated molar concentration of carbon in the samples obtained from the nominal solution concentration and known % m/m of C in the respective samples determined via elemental analysis (Table S1)." Raman scattering of pure water? As an emission standard? If it is Raman scattering from each sample, this can not be used as an internal standard. The reason for normalisation per C% are unclear.

The authors use quinine sulfate as emission standard. This compound has QY close to 100%, and absorbs in the UV, and is not the best standard for the measurements.

4. The emission measurements also show strong emission overlapping with absorption. This observation implies that completely different entities emit at different parts of the spectrum, and that there is no energy transfer between different emitting species. This is unusual. Emission spectra need to be remeasured under correct optical conditions. There is a discussion about the threshold for when energy transfer start taking place (at wavelengths longer than a "localization threshold") but this discussion is too broad.

5. The ultrafast TA study is self-consistent and the data analysis is sound. The observation of hole-burning confirms that the ground state spectra are superposition of individual chromophores which do not interact with one another. These data are reliable as optical densities used are <0.2. The explanation involving delocalization threshold for energy transfer is also plausible. This part can be published on its own. The fit residuals in Fig S6 show considerable deviation at early times.

6. The discussion about few-nm structures as determining the overall properties, as well as formation of charge-transfer excitons and exciton-delocalisation discussion are interesting, and plausible. This is one of the conclusions of the article which is supported by the discussion and the data.

Overall, the manuscript suggests a generalized mechanism of light absorption, photophysical properties and photoprotection as arising from aggregation of flat chromophoric units to a size of a few nanometers. This is probably correct for melanin and derivatives, and has long-ranging consequences for both the understanding of how such materials work and how to design new materials.

However, the manuscript attempts to build a unified picture of properties of all materials which have unstructured absorption across visible region. Perhaps if the discussion is more focussed this work can be reconsidered for publication.

Author's Response to Peer Review Comments:

We appreciate the thoughtful and thorough comments of the reviewers, which helped us to correct a few errors and to communicate our arguments and conclusions with greater clarity as described in the attached response file.

Point-by-point Response to Reviewers

Unless otherwise indicated, page and figure numbers in our responses refer to the revised text. We appreciate the though<ul and thorough comments of the reviewers, which helped us to correct a few errors and to communicate our arguments and conclusions with greater clarity as described in detail below.

Reviewer 1

Comment: *In their manuscript entitled "Common Photoproperties of Eumelanin and Natural Organic Matter Emerge from Ensembles of Few-Layered Nanostructures", McKay, Kohler and coworkers report how basic photoproperties that include transient spectral hole burning and excitation-wavelength dependent emission arise in melanin and natural organic matter from the ensemble behaviour of these ultrasmall units. From the measurements presented in this manuscript, the authors propose that NOM and synthetic melanin share closely related optical properties but lie along a morphological gradient. DOPAm becomes substantially more NOM-like when disassembled into smaller units, emphasizing the sensitivity of carbon-rich nanomaterials to electronic interactions. Overall, the manuscript presents very high-quality*

measurements to support the claims made that are central to the understanding of the optical properties of eumelanin. The manuscript is recommended for publication in the present form.

Authors' Reply: We thank the reviewer for the positive evaluation of the manuscript and for highlighting that the conclusions are well supported by high-quality measurements.

Reviewer 2

Comment: *Many photophysical studies of melanin and related natural materials have been reported. I suspect even more have been attempted and abandoned. The problem is that the absorption is broad and unstructured, making it diMicult to pinpoint what species are absorbing the light. This paper established a foundational viewpoint for future studies. Not only does the work provide an appealing testable model for eumelanin photophysics, but it established a unified framework for comparable materials. The paper focuses in that regard on natural organic matter, but the work will also inspire new carbon dot research.*

I had several questions as I read the paper, but it turned out that they were nicely addressed in page 25 and beyond. The importance of aggregates appeared to be incompatible with the observed lack of energy transfer, but that was addressed. I like the disorder density of states model, Fig 4, from Heinz Bässler, it is a key advance of the work to base the model around this picture. Indeed, that same picture proved important for understanding disorder in conjugated polymers. I was also wondering about CT states, but, again, this is addressed. A related important comment in the work is that substitution of these small molecules can have a profound influence on the photophysics.

Overall, I think this paper has potential to be highly influential. It should be published.

Authors' Reply: We thank the reviewer for the very positive assessment of our work.

Reviewer 3

General Comment: *The authors use spectroscopy to demonstrate similarity at the nanoscale leading to similar photophysical properties between synthetic melanin and natural organic matter. The data are convincing, the manuscript is well-written and clear, and the implications for future research are interesting. My only suggestion is that the authors include some measurement of natural melanin, or an explanation of why use of synthetic melanin is justified here, given that the point of the paper is to compare two natural forms of melanin. If they argue that structure of synthetic melanin is close enough to natural melanin for use here, they should clearly explain why.*

Authors' Reply: We thank the reviewer for thoroughly evaluating the manuscript and for their positive assessment of the data, clarity, and broader implications of the work.

Comment 1: *P5L3: I would not attribute a "functional role" to NOM.. this implies (as does "allow") that it has evolved to perform this role when of course it is non-living material that does not evolve. "their similar properties cause melanin pigments and nom to have similar eMects" might be a better way to phrase it*

Authors' Reply: We agree with this point and have revised the text.

> *Changes are highlighted in yellow*
> [p. 5] Their similar properties allow melanin pigments and NOM to act analogously in their respective environments.

Comment 2: *P8L17: Since the paper is framed as a comparison between the mechanisms of natural melanin and NOM, it seems less than optimal to use synthetic melanin. Why not use sepia or some other natural melanin source?*

Authors' Reply: This is a valid question, which we believe will occur to readers. For decades, synthetic melanins have been studied as surrogates for natural melanins because they overcome several challenges of working with the latter material. Natural melanins occur in a range of morphologies that depend on the biological source and extraction protocol, contain variable amounts of metal ions and proteins, and are notoriously diDicult to solubilize. In contrast, synthetic melanins can be prepared reproducibly and oDer greater control over particle morphology. Synthetic DOPA melanin is a widely used model system for natural eumelanin, as it is proposed to contain similar chemical structures and hierarchical motifs as the natural pigment (Watt et al., *Soft Matter*, **2009**, *5*, 3754).

We have added the following text to the introduction to clarify this rationale.

> [p. 8] Synthetic melanins o?er greater reproducibility and control over morphology compared to natural melanin, which can vary significantly depending on the source, method of isolation, and the presence of residual proteins and bound metal ions. DOPAm is widely used as a model for natural eumelanin and is thought to capture its key chemical motifs and hierarchical structures.

Reviewer 4

General Comment: *The manuscript presents an interesting study focussed on apparent similarities between melanin and derivatives, and "molecular organic matter" which is a broad definition of multiple compounds and mixtures thereof arising from decomposition of organic matter.*

The authors hint at the potential for unified formalism to explain properties of all nanomaterials. This would be very important.

The authors' main point is "parallel photophysics of melanin and NOM."

It is diMicult to understand what is meant by "parallel photophysics", but from the context of the article this statement may refer to the similarities of photophysical properties which in the authors' view originate from similar morphologies.

The last sentence of the introduction states that "Taken together, this work advances understanding of how the optical properties are tuned by particle morphology and identifies length scales at which key optical properties emerge."

This sentence can be applied to a very broad range of materials and is not specific. The authors use AFM, steady-state absorption and emission spectroscopy, and time-resolved spectroscopy to investigate the properties of two groups of substances.

It is stated that "NOM and melanin exhibit remarkably similar steady-state and transient photo-properties, with some second order diMerences. " It is not clear what "second-order diMerences" refer to. Further, the data presented show the similarities which appear to be purely coincidental.

There are multiple materials (for example, molecular organic nanosheets) which would exhibit the same properties.

Whilst the manuscript raises interesting questions about serendipity in Nature, and as such may be of interest to the broad readership of any journal, the scientific arguments presented in the paper do not support the physico-chemical claims of the similarities of the properties. It is diMicult to follow what the key argument is – is this that when molecules pack together in nanostructures (the authors use the term "ultrasmall" without quantifying it), they have similar optical properties? This can not be universally correct. The evidence presented does not support the claim of fundamental similarities, but rather coincidental properties. Further, the work on the analysis of steady-state spectra raises serious concerns (see below).

Thus, despite a list of interesting observations, it is not possible to support publication of this study. The ultrafast spectroscopy part is of high quality and could be potentially published separately.

Detailed comments are given below.

Authors' Reply:

We thank the reviewer for their comments. The points raised concern the key argument, the scope of the claims, the interpretation of similarities between NOM and melanin, and the clarity of terminology. We address these points below.

Key argument of the study: The central claim of the manuscript is presented in the Introduction as follows (yellow highlighting shows changes made to the original text): Using AFM imaging, we show that the DOPAm and NOM samples contain abundant few-layer stacks of planar molecules, and we propose that these ultrasmall assemblies define the elementary chromophores whose ensemble-level behavior gives rise to their common photophysical properties.

Scope and intent of the study: We clarify that we are not proposing a universal formalism for *all* nanomaterials. Rather, our intent is to lay the groundwork for a unified photophysical framework for NOM and melanin that may be extended to related disordered carbon nanomaterials. This study is motivated by the observation that many carbon-based materials share a common set of properties listed in Figure 1a, including broadband absorption, excitation-dependent emission and redox activity, yet clear structure-property relationships in these systems remain elusive due to their high chemical heterogeneity. The present work examines NOM and a synthetic eumelanin together, focusing specifically on their photoproperties, to gain insight into the structural origins of their photophysics. To avoid overgeneralization, we have revised the final sentence of the Introduction to explicitly reference the two systems studied here, NOM and melanin:

> [p. 8] Taken together, this work advances understanding of how the optical properties of eumelanin and NOM are tuned by particle morphology and identifies length scales at which key optical properties emerge.

Similarities are not coincidental: The reviewer raises the concern that similarities observed across steady-state and ultrafast spectroscopic measurements are coincidental. We note that the qualitative similarities between NOM and eumelanin can be tuned and brought into closer quantitative agreement through nanostructural modification. Disassembly of DOPAm leads to photoproperties that are closer to those of NOM, demonstrating that these similarities are not coincidental but are governed by nanoscale organization.

Clarification of terminology:

(i) Ultrasmall: Nanoparticles are generally understood to have sizes between 1 and 100 nm. We use ‘ultrasmall’ to identify nanoparticles that fall on the smaller end of this range. Specifically, we refer to few-nanometer size nanoparticles as ultrasmall, consistent with literature usage (see Epple et al., *Acc. Chem. Res.* **2023**, *56*, 3369). We modified the text on p. 8 to define “ultrasmall.”

[p. 8] ...the synthetic melanin nanoparticles into ultrasmall (< 5 nm) few-layered structures that closely mimic the optical properties of NOM samples…

(ii) Parallel photophysics: By "parallel photophysics," we mean that melanin and NOM exhibit similar photophysical behaviors. To avoid ambiguity, we replaced "parallel" with "common" in the following text.

[p. 9] chromophores whose ensemble-level behavior gives rise to their common photophysical properties.

(iii) Second-order diDerences: While NOM and melanin exhibit qualitiatively similar steadystate and ultrafast photophysical behavior (e.g., broadband absorption, excitationwavelength-dependent emission, broad photoinduced absorption, and transient spectral hole burning), quantitative diDerences are observed in absorption spectral slopes, fluorescence quantum yields, transient absorption spectral weighting below 350 nm, and excited-state lifetimes. These diDerences are described in detail in Section 2.1, and the following changes have been made to replace the phrase "second-order diDerences".

[p. 9] Despite these molecular-level di?erences, NOM and eumelanin exhibit qualitatively similar steady-state and transient photoproperties as presented in this section. Nonetheless, quantitative di?erences in properties such as fluorescence quantum yields and excited-state lifetimes indicate that their common properties occur on a continuum. In later sections, we explore the hypothesis that these di?erences are tuned by nanoscale interactions among chromophores.

[p. 19] These observations indicate that the photoproperties of DOPAm are sensitive to nanostructure. We propose that di?erences in the photophysical properties of DOPAm and NOM narrow when they acquire similar morphologies.

Comment 1: *"The electronic absorption spectra of both DOPAm and the NOM samples are broad, featureless, and decrease monotonically from UV to NIR wavelengths (Figure 2a). The spectra decay exponentially with wavelength such that a graph of log absorbance vs. wavelength yields an approximately straight line."*

There are multiple (potentially unlimited) number of molecules and materials that exhibit a long "tail" absorption across visible range and into NIR. This is usually attributed to a number of individual electronic transitions, at slightly diMerent energies due to slightly diMerent environments. This is typical for aggregates of light-absorbing molecules and is often observed when aggregates of multiple compositions are formed (e.g. dimer, trimers, tetramers etc existing in the same solution). Unless there is a specific physical (chemical) reason for the stated log dependence, it can also originate from scattering. The log dependence appears overinterpreted, and a careful examination of Fig S1 indicate that the dependence is not linear. Any dependence of the absorbance should relate to the energy of the transition not the

wavelength (unless this is a discussion of quantum confinement). It is possible that – as in carbon-based nanomaterials – there is a distribution of band-gaps causing the continuous absorption feature. But this in itself does not mean that melanin and the decomposition products are similar in properties. (In perhaps a crude analogy, there are multiple compounds that absorb around 400 nm. This does not imply that their properties are similar or related).

<u>Authors' Reply:</u> The reviewer raises two main concerns about (1) the origin and interpretation of the approximately exponentially decaying absorbance vs. wavelength and (2) the scope of the similarities in properties claim. We address these concerns in the points below:

Interpretation of log-linear behavior: Fitting the absorption spectrum to a function that decays exponentially with increasing wavelength is a widely used empirical metric in both the melanin (e.g., K. C. Littrell et al., *Photochem. Photobiol.* **2003**, *77*, 115; P. Meredith et al., *Soft Matter*, **2006**, *2*, 37) and NOM literature (e.g., A. Bricaud et al., *Limnol. Oceanogr.* **1981**, *26*, 43; R. Del Vecchio and N. V. Blough, *Environ. Sci. Technol.* **2004**, *38*, 3885; B. P. Yakimov et al., *Environ. Sci. Technol.* **2021**, *55*, 10365). The reviewer is correct that a graph of log "absorbance" is not perfectly linear vs. wavelength—slight deviations can also be seen in Figure 2 of Littrell 2003 and Figure 1a of Yakimov 2021. Also, lineshape models in molecular spectroscopy are often expressed more simply in terms of frequency, but our aim is neither to explain the approximately linear decay of log absorbance vs. wavelength nor to interpret the meaning of the slope parameter reported in Table S3. Instead, our intent is to highlight a common descriptive framework, which like other concepts noted in our paper arose independently in the two fields.

The reviewer's comment next addresses *why* the absorption spectrum has this characteristic. This question has been posed in both fields for decades, but a satisfactory answer is still elusive. The reviewer's point about light scattering has been raised in both the melanin and NOM fields but has been ruled out. Measurements on a synthetic eumelanin very similar to our DOPA melanin indicate that scattering contributes < 6% to total attenuation even at UV wavelengths from 210 nm – 325 nm, where the strongest scattering is observed (J. Riesz et al., *Biophys J.* **2006**, *90*, 4137–4144). It is correct that Rayleigh scattering by particles with diameters up to approximately 10% of the wavelength, λ, varies as "!! (which is nevertheless distinct from $\exp(-S\lambda)$), but this will determine the shape of the attenuation spectrum only in regions where there is negligible absorption. The (small) deviations that we observe from a straight line do not match the trend observed for turbid ocean waters (Figure 2 in Bricaud et al.1981), indicating that the scattering contribution from our NOM samples is low, as expected for isolated humic substances reconstituted in water. In summary, light scattering contributes negligibly to the shape of our spectra, justifying the labeling of the spectra by absorbance in Figure 2a and Figure S1.

The scope of similarities in properties claim: We agree that similar absorption spectra alone do not imply broader similarities in physicochemical properties. We are careful to use phrases like "common photoproperties", "common photophysical properties", "common photophysics", etc. in the paper's title and throughout the text. We corrected a few instances where 'properties' was used without a qualifier:

> [p. 6] Second, we advance the hypothesis that morphology and hierarchical structure are paramount for photoproperty emergence in melanin and NOM, pointing out the opportunity for a nanoscience perspective to advance understanding.
> [p. 26] The NOM-like photoproperties of the LMW fraction suggest that the photophysical changes only emerge…
> [p. 30] DOPAm becomes substantially more NOM-like in optical properties when disassembled…

<u>Comment 2:</u> *Fig 2 FTIR – the full spectrum is presented, which has some groups of interest identified. These are not labelled, and whilst they are likely be (CO) groups, amide groups, and -CH, this figure needs to be much clearer.*

<u>Authors' Reply:</u>

Although some investigators have attempted to assign bands in IR absorption spectra of various melanins, these assignments are unreliable given that the actual chemical structures present in these heterogeneous materials are uncertain. Furthermore, the fact that a particular local mode, such as a carbonyl stretching vibration, can be tuned by many tens of cm^{-1} as a result of conjugation or other changes to its local environment points out the futility of confidently making vibrational assignments based on typical frequencies of organic functional groups. The purpose of Figure 2c is not to provide assignments but to highlight the pronounced diDerences between the FTIR spectra of NOM and DOPAm. As stated in the last paragraph on p. 9, this indicates atomic level structural diDerences. To avoid over-interpretation of the individual bands in the spectra, we have removed the gray dashed lines from Figure 2c.

<u>Comment 4:</u> *Figure 2d,e,f is unclear. The black dots represent some shift of the emission maximum? What does the white spectrum underneath the emission spectra represent?*

Author's Reply: As stated in the caption, the black dots represent the emission maximum of each emission spectrum. This illustrates how the emission maximum shifts to longer wavelengths as the excitation wavelength is tuned to longer wavelengths. We clarify now that the black dot shows the intensity-weighted emission maximum calculated as

where F(is the fluorescence intensity measured at wavelength λ(

λ%&’#$ = *F(λ(/-*F(.
((

. We added the definition to SI section S5 and added a clarification to the main text:

> [p. 10] Likewise, the maximum wavelength of the emission band (λ#$%!" calculated as the intensity-weighted emission maximum, see SI section S5), (black circles in Figures 2d–f, S2b) …

By ‘white spectrum’, we think the reviewer is referring to the white area near the wavelength axis (circled below for Figure 2e as an illustration). The detection window for each emission spectrum moves progressively to longer wavelengths when the excitation wavelength is increased because emission occurs at wavelengths longer than the excitation wavelength. This leaves an (empty) white area that is structured but that has no significance. It simply results from where the colored emission curves start and stop. Similar structures in a white background are seen in Figure 1b,c in Yakimov et al., *Environ. Sci. Technol.* **2021**, *55*, 10365.

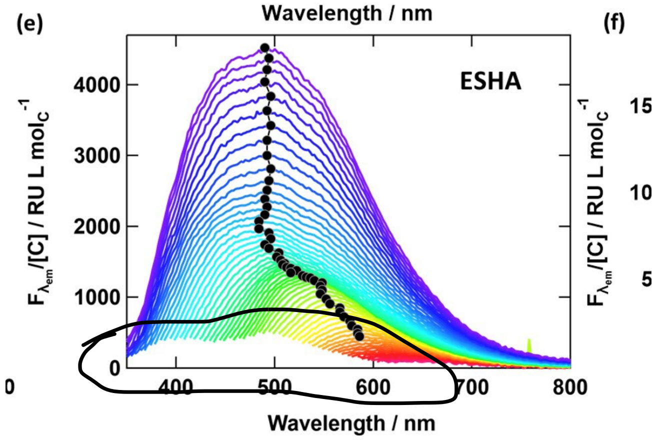

Comment 5: *The dependence of QY (Fig 2b) on the excitation wavelength requires further analysis. The absorption spectra presented are normalised. Thus it is not clear which optical density was used to record each emission spectrum The authors state (just above Fig S1 in the SI) that they corrected for inner-filter eMect. They also state the optical densities for emission measurements were up to 1.5. It is therefore not possible to agree that the measurements have been done correctly.*

Author's Reply: The total optical density at the excitation and emission wavelength ($A_{)"\#} +$ $A_{)"\$}$) was kept at less than 1.5 as measured in a 1 cm cuvette because prior research has demonstrated that absorbance-based inner filter corrections are eDective at or below this value (Kothawala et al., *Limnol. Oceanogr.: Methods*, **2013**, *11*, 616) In response to this comment, the text in SI section S5 has been revised to more clearly state that the 1.5 refers to the total optical density at the excitation and emission wavelengths.

> [p. S8 of SI] To ensure that inner filter corrections remained e?ective, the sum of absorbance at the excitation and emission wavelength was kept at less than 1.5 as measured in a 1 cm cell ($A_{\&!"} + A_{\&!\#} \leq 1.5$).

Comment 6: *The authors describe that "Fluorescence intensities were normalized to the Raman scattering unit area (measured at a 350 nm excitation wavelength) and were further normalized to the calculated molar concentration of carbon in the samples obtained from the nominal solution concentration and known % m/m of C in the respective samples determined via elemental analysis (Table S1)." Raman scattering of pure water? As an emission standard? If it is Raman scattering from each sample, this can not be used as an internal standard. The reason for normalisation per C% are unclear.*

Author's Reply: Fluorescence intensities were normalized to the Raman scattering unit area in pure water (clarification added to SI section S5), measured daily. Normalization to carbon concentration, although not completely necessary for understanding the data, is a common practice in the NOM field to account for variations in carbon content across diDerent samples.

> [p. S8 of the SI] Fluorescence intensities were normalized to the Raman scattering unit area of pure water (measured at a 350 nm excitation wavelength)

Comment 7*: The authors use quinine sulfate as emission standard. This compound has QY close to 100%, and absorbs in the UV, and is not the best standard for the measurements.*

Author's Reply: Ideally, a fluorescence standard will absorb at all wavelengths for which QYs are calculated. Quinine sulfate does not absorb at wavelengths > 400 nm, which are the excitation wavelengths of interest for NOM and DOPAm fluorescence. Thus, the quantum yields of the organic matter samples were calculated relative to quinine sulfate absorbance and fluorescence at a fixed excitation wavelength of 350 nm. This approach has been used before in both the NOM (Del Vecchio and Blough, *Environ. Sci. Technol.* **2004**, *38*, 3885) and melanin (Nighswander-Rempel et al., *J. Chem. Phys.* **2005**, *123*, 194901) fields and is justified by the use of a reference detector and instrument-specific correction factors. Measuring the fluorescence intensity in signal-over-reference (S/R) mode normalizes the fluorescence intensity observed to diDerences in incident intensity. Instrument-specific correction factors

correct for diDerences in light transmission through the optical components (e.g., monochromators) at diDerent excitation and emission wavelengths.

<u>Comment 8:</u> *The emission measurements also show strong emission overlapping with absorption. This observation implies that completely diMerent entities emit at diMerent parts of the spectrum, and that there is no energy transfer between diMerent emitting species. This is unusual. Emission spectra need to be remeasured under correct optical conditions. There is a discussion about the threshold for when energy transfer start taking place (at wavelengths longer than a "localization threshold") but this discussion is too broad.*

<u>Authors' Reply:</u>

The optical conditions and corrections applied to the emission measurements are appropriate as discussed in the previous response. We agree that the absence of excitation energy transfer (EET) is unusual (for a mixture of non-interacting molecules), but we emphasize that this behavior is well documented in both melanin (Nighswander-Rempel et al., *J. Chem. Phys.* **2005**, *123*, 194901) and NOM (Del Vecchio and Blough, *Environ. Sci.*

Technol. **2004**, *38*, 3885). As noted by the reviewer in their next comment, the ultrafast spectral hole-burning measurements further support restricted energy transfer under visible excitation, as evidenced by the absence of spectral shifting of the bleach over time.

While the reviewer does not state any specific issues with the restricted EET model, they are raising concern about the broadness in the scope of applying this model to all aggregated materials in the context of their previous and next comments, if we understand correctly. We would like to clarify that we apply the model of localization threshold only to NOM and melanin. The excitation dependent emission behavior mirrors site-selective fluorescence observed in conjugated polymers, where energy transfer is suppressed upon excitation at the red edge of the absorption spectrum. Therefore, we adapt the well-established concept of site-selective fluorescence in conjugated polymers to interpret our observations in NOM and melanin. This framework explains the emission and transient spectral hole burning results supporting the presence of a localization threshold (no EET below a threshold energy and restricted EET above the threshold).

<u>Comment 9:</u> *The ultrafast TA study is self-consistent and the data analysis is sound. The observation of hole-burning confirms that the ground state spectra are superposition of individual chromophores which do not interact with one another. These data are reliable as*

optical densities used are <0.2. The explanation involving delocalization threshold for energy transfer is also plausible. This part can be published on its own. The fit residuals in Fig S6 show considerable deviation at early times.

Authors' Reply: We thank the reviewer for the positive assessment of the ultrafast TA study and the data analysis. We note that the steady-state measurements have been carried out with appropriate and eDective corrections as described above. Furthermore, trends such as fluorescence QYs that decrease with increasing excitation wavelength (despite the decreasing sample absorbance) agree well with previous reports on NOM (see, for example, ref. 11), validating our corrections. With respect to the fit residuals in Fig. S6, the apparent deviations at early times arise primarily from the way the data were graphed. In the original figure, the residuals were plotted on a linear time axis, while the transient absorption measurements are sampled much more densely at early delay times where the signals change most rapidly. The resulting crowding of the data points in the early-time region made the residuals appear larger. We have re-plotted the residuals on a logarithmic time axis. In addition, we have adjusted the fitting window to begin at 0.6 ps rather than 0.4 ps to avoid artifacts caused by the instrument response at the earliest delay times. Now, the residuals behave similarly at all delay times. The new fitting parameters shown in Table S3 diDer insignificantly from the previous ones and do not change our previous conclusions.

Comment 10: *The discussion about few-nm structures as determining the overall properties, as well as formation of charge-transfer excitons and exciton-delocalisation discussion are interesting, and plausible. This is one of the conclusions of the article which is supported by the discussion and the data.*

Overall, the manuscript suggests a generalized mechanism of light absorption, photophysical properties and photoprotection as arising from aggregation of flat chromophoric units to a size of a few nanometers. This is probably correct for melanin and derivatives, and has long-ranging consequences for both the understanding of how such materials work and how to design new materials.

However, the manuscript attempts to build a unified picture of properties of all materials which have unstructured absorption across visible region. Perhaps if the discussion is more focussed this work can be reconsidered for publication.

Authors' Reply:

We would like to clarify that this study develops a framework based on ensembles of fewnanometer-sized chromophores specifically for NOM and eumelanin (see wording in the abstract), which may be extended to related carbon-based materials in the future. We revised a sentence in the conclusions section to make it clearer that the framework applies to NOM and melanin but is likely to be valuable for other carbon-based nanomaterials:

[p. 31] Because π-stacking and hydrogen-bonding motifs recur across disordered carbon nanomaterials, our approach suggests that the framework developed here for NOM and eumelanin may be valuable for elucidating the photophysics of other carbon-based materials.
